# Supplementary material for: Promoting H2O2 production via 2-electron oxygen reduction by coordinating partially oxidized Pd with defect carbon
Source: Nat Commun. 2020 May 1;11:2178. doi: 10.1038/s41467-020-15843-3 (PMC7195490; doi:10.1038/s41467-020-15843-3)
Supplement: Supplementary file 1 — Supplementary information [file 41467_2020_15843_MOESM1_ESM.pdf]

Supplementary Information

Promoting H_2O_2 Production via 2-electron Oxygen Reduction by Coordinating Partially Oxidized Pd with Defect Carbon

Chang *et al.*

Supplementary Figures

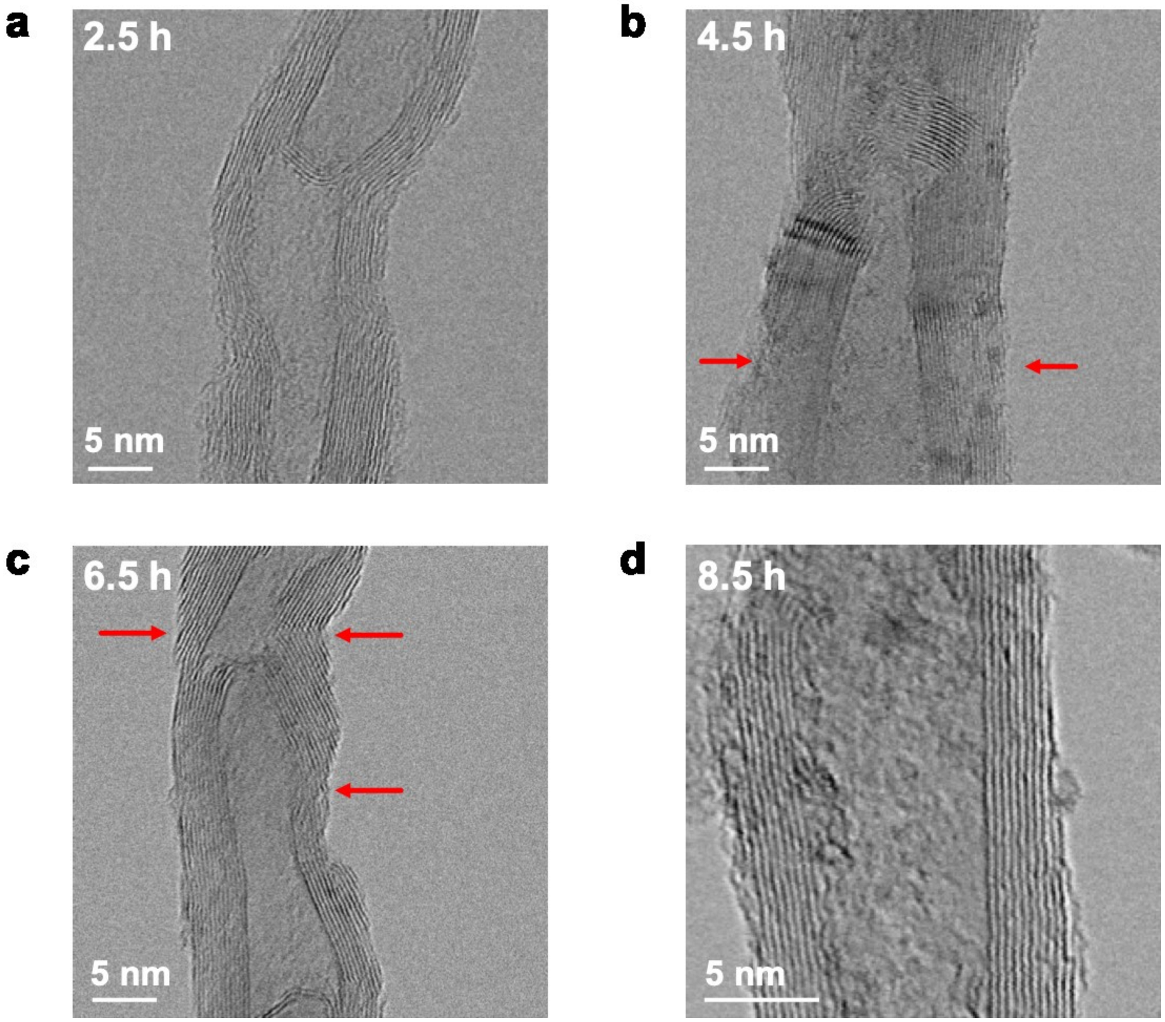

Supplementary Figure 1. HRTEM image of obtained OCNT for different reaction time **a** 2.5 h, **b** 4.5 h, **c** 6.5 h, **d** 8.5 h.

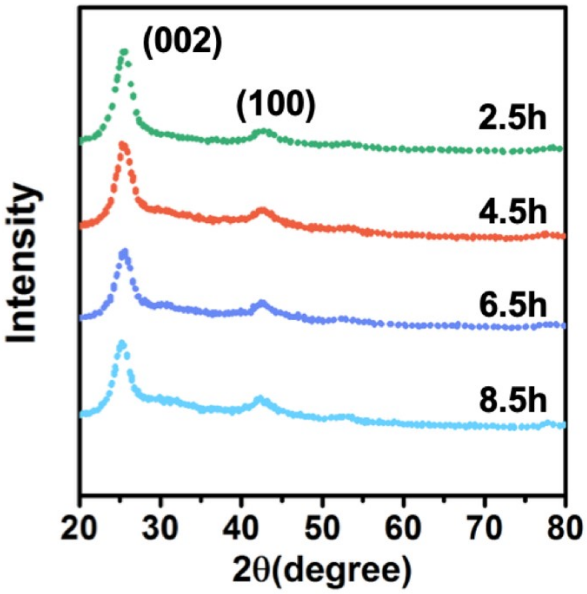

Supplementary Figure 2. XRD patterns of obtained OCNT samples with different durations of oxidation time.

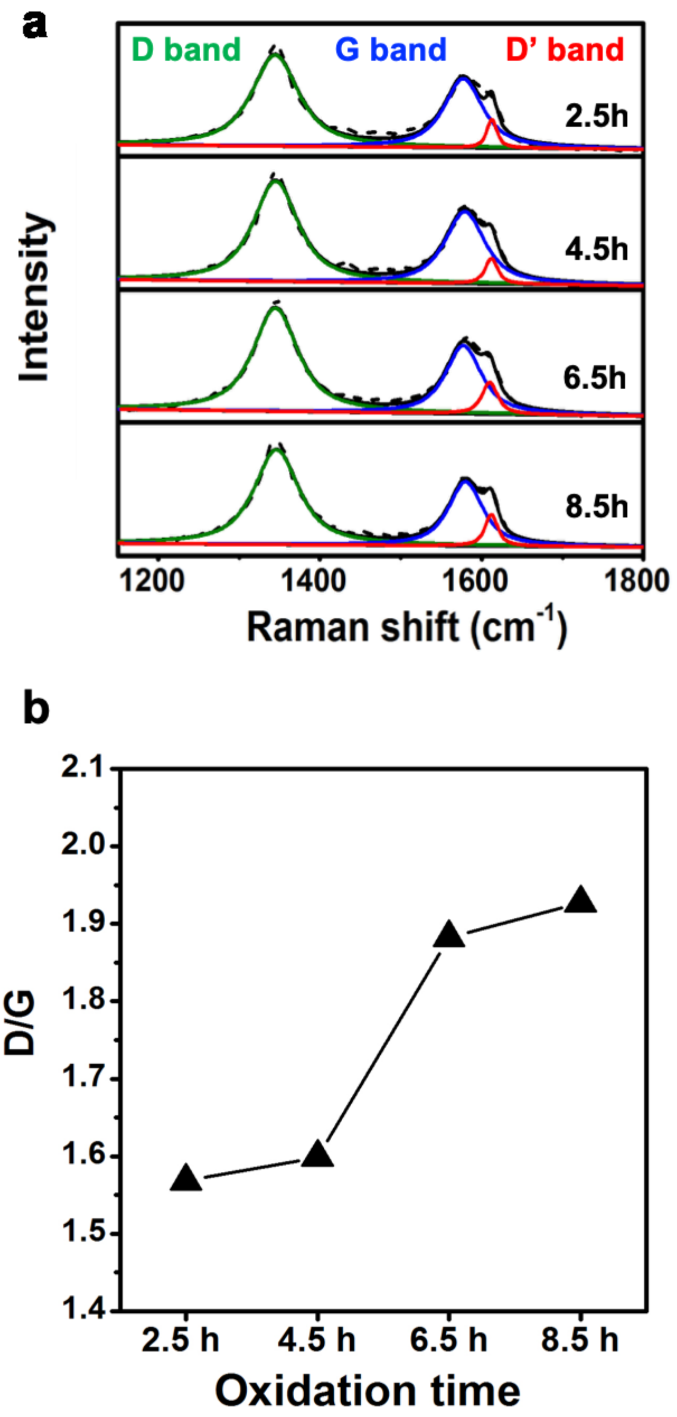

Supplementary Figure 3. **a** Raman spectra and **b** calculated I_D/I_G ratios of different OCNT samples.

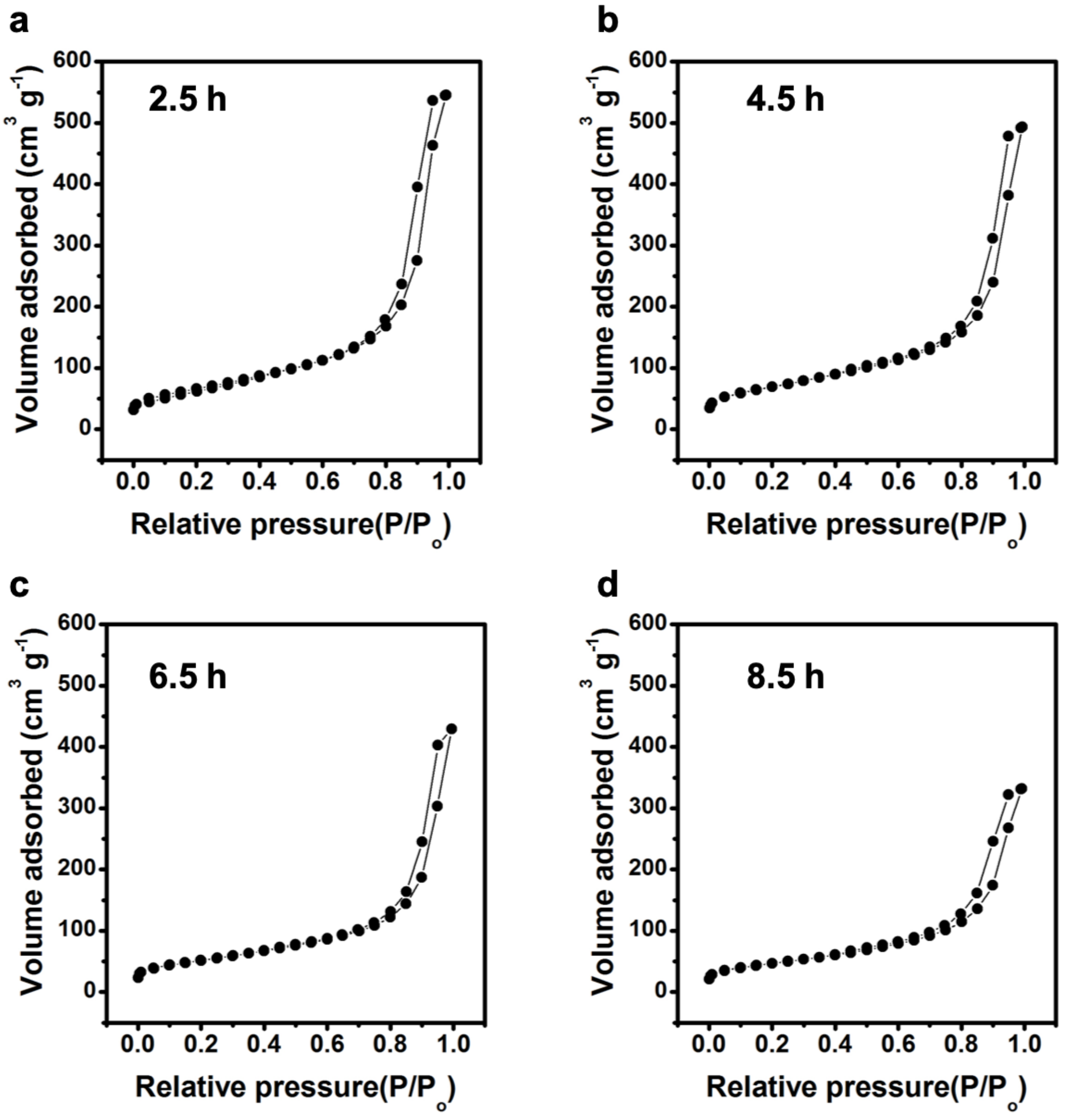

Supplementary Figure 4. The N_2 adsorption-desorption isotherms of OCNT samples. **a** 2.5h OCNT, **b** 4.5h OCNT, **c** 6.5h OCNT, **d** 8.5h OCNT.

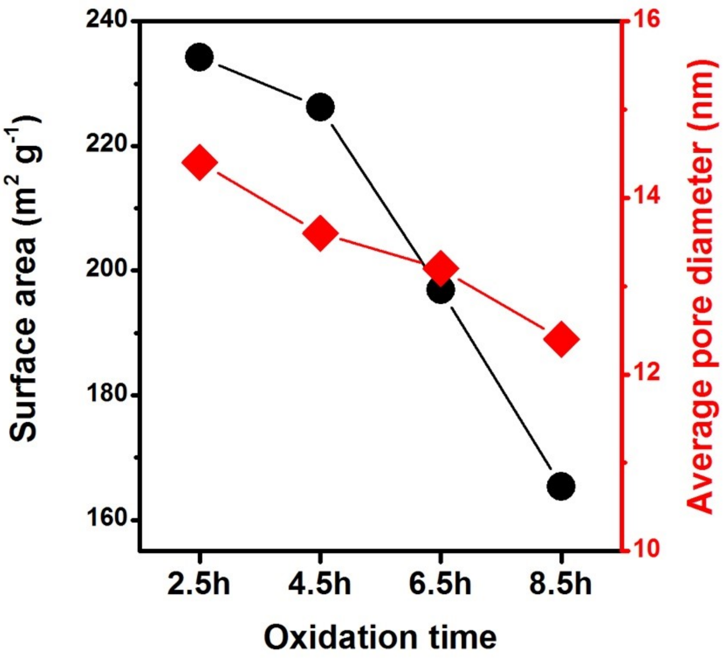

Supplementary Figure 5. Calculated surface area and average pore diameter from N_2 adsorption-desorption isotherms of OCNT samples.

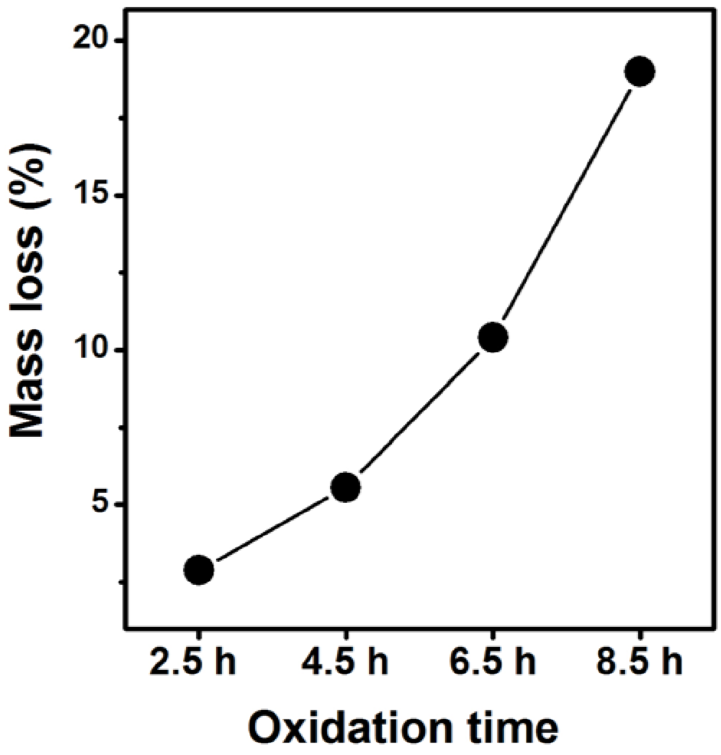

Supplementary Figure 6. Recorded mass loss percentages during the preparation of OCNT samples.

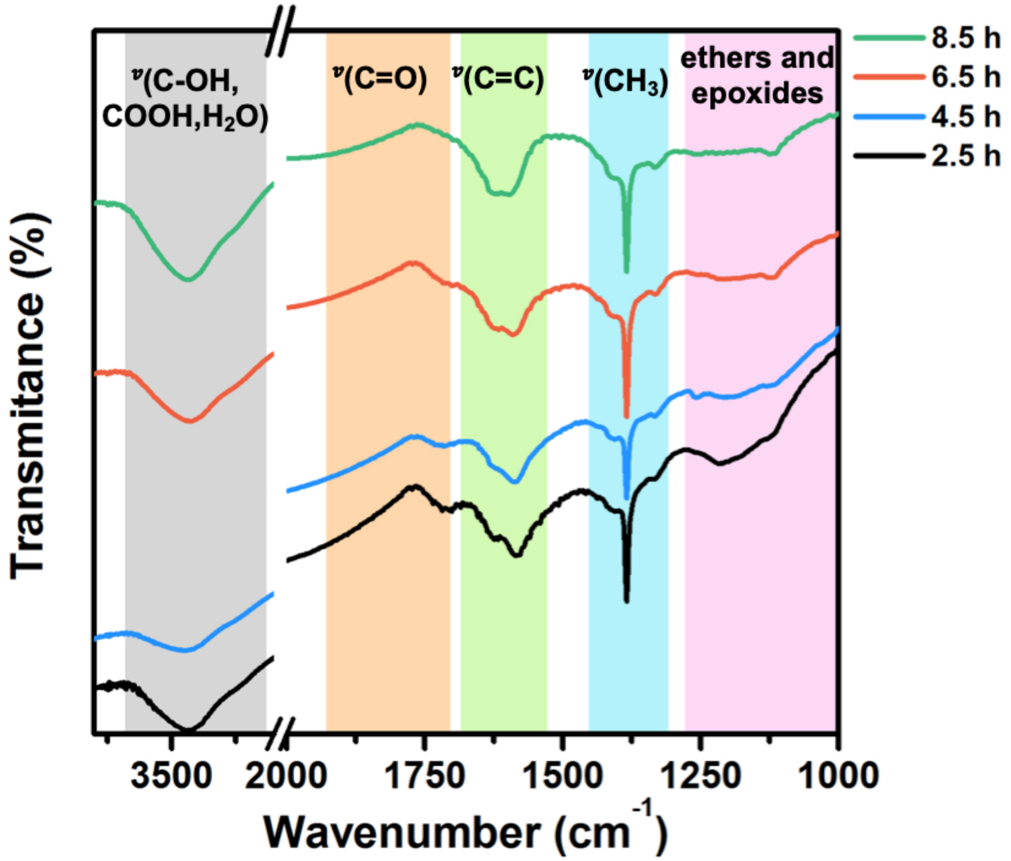

Supplementary Figure 7. FTIR spectra of OCNTs prepared from 2.5 h to 8.5 h. Peak assignments are as follows: hydroxyls (broad peak at 3050–3800 cm^{-1}), C=O (1700–1900 cm^{-1}), C= C (1500–1600 cm^{-1}), CH_3 (1375 cm^{-1}) and ethers and epoxides (1000–1280 cm^{-1}).

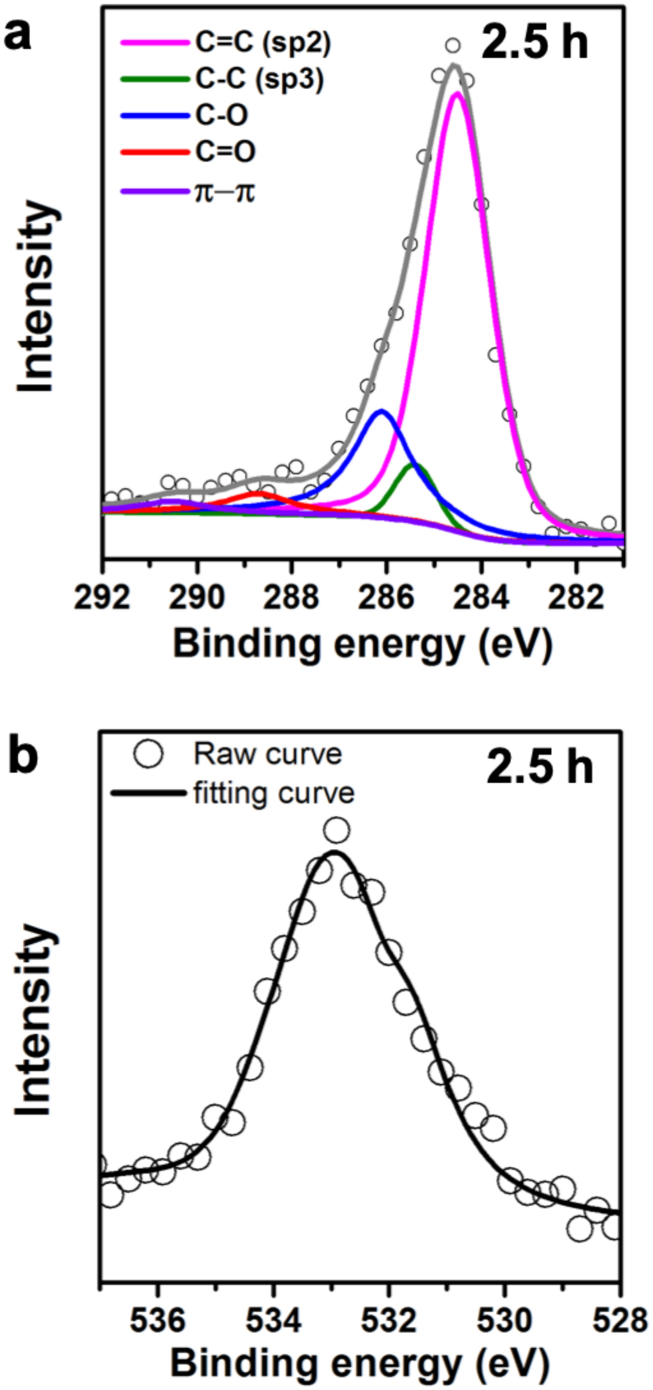

Supplementary Figure 8. **a** Deconvoluted carbon 1s spectra and **b** obtained oxygen 1s spectra of OCNTs reacted for 2.5 h.

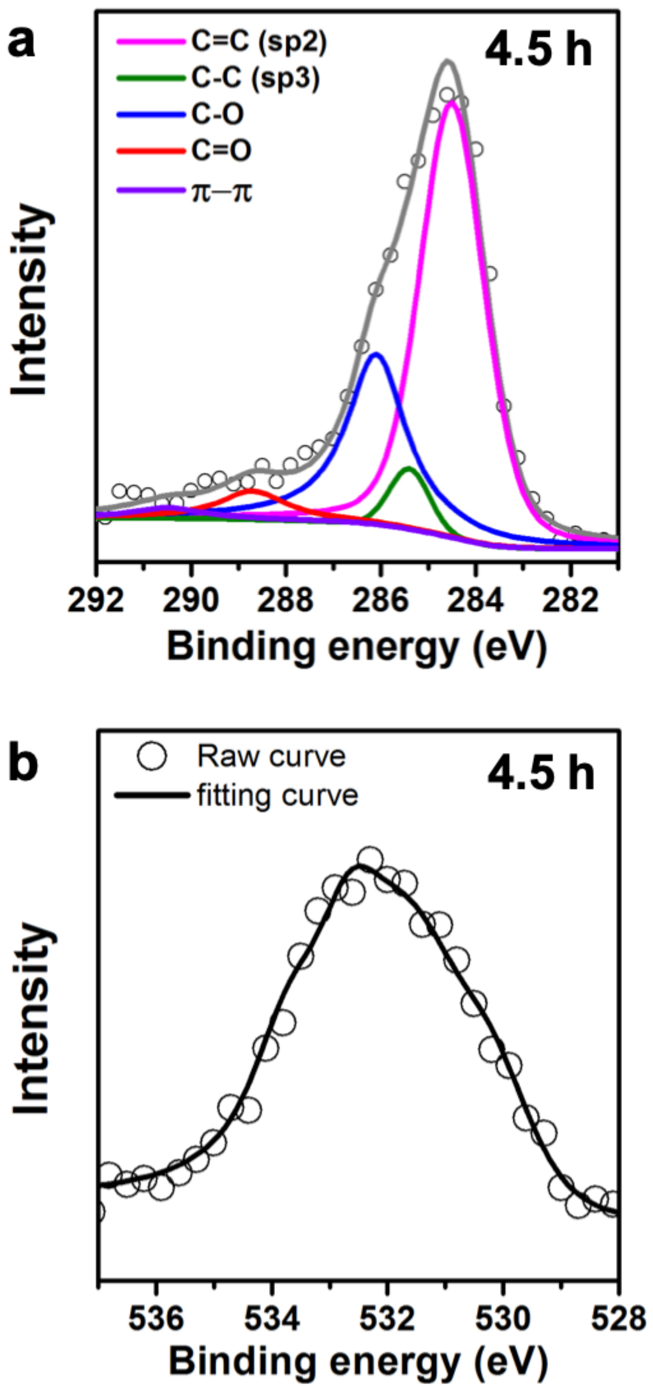

Supplementary Figure 9. **a** Deconvoluted carbon 1s spectra and **b** obtained oxygen 1s spectra of OCNTs reacted for 4.5 h.

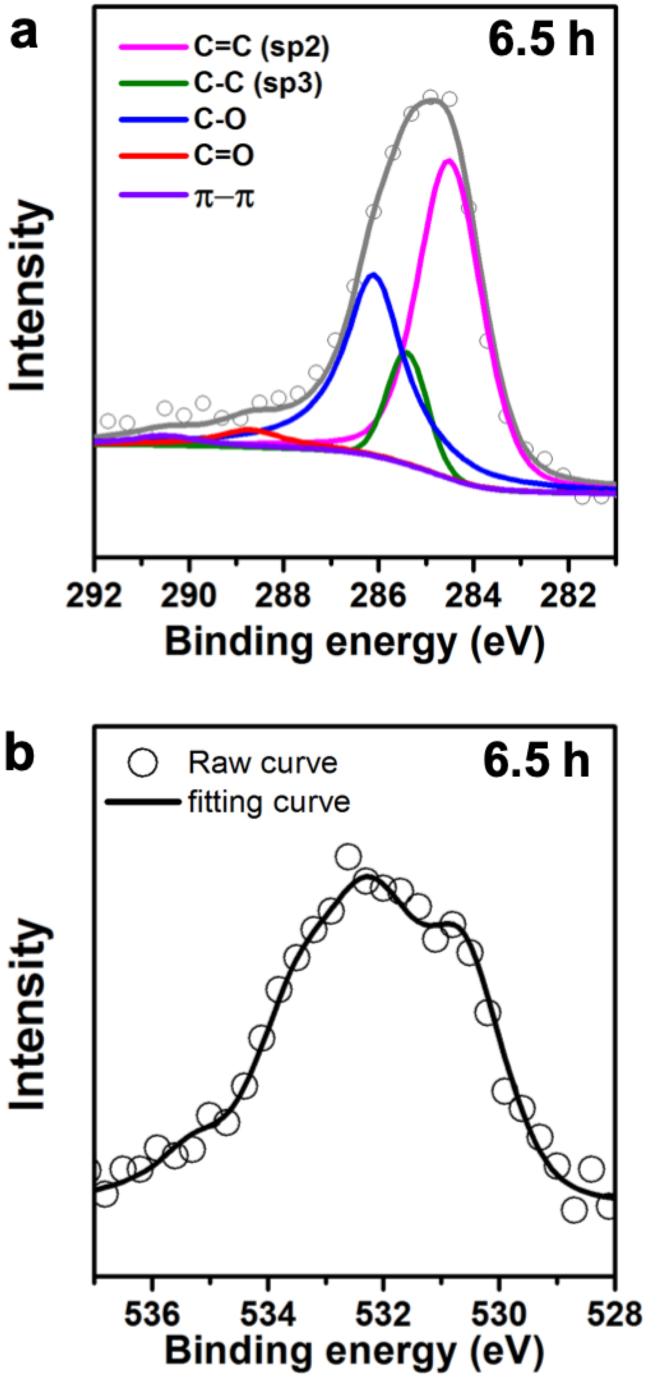

Supplementary Figure 10. **a** Deconvoluted carbon 1s spectra and **b** obtained oxygen 1s spectra of OCNTs reacted for 6.5 h.

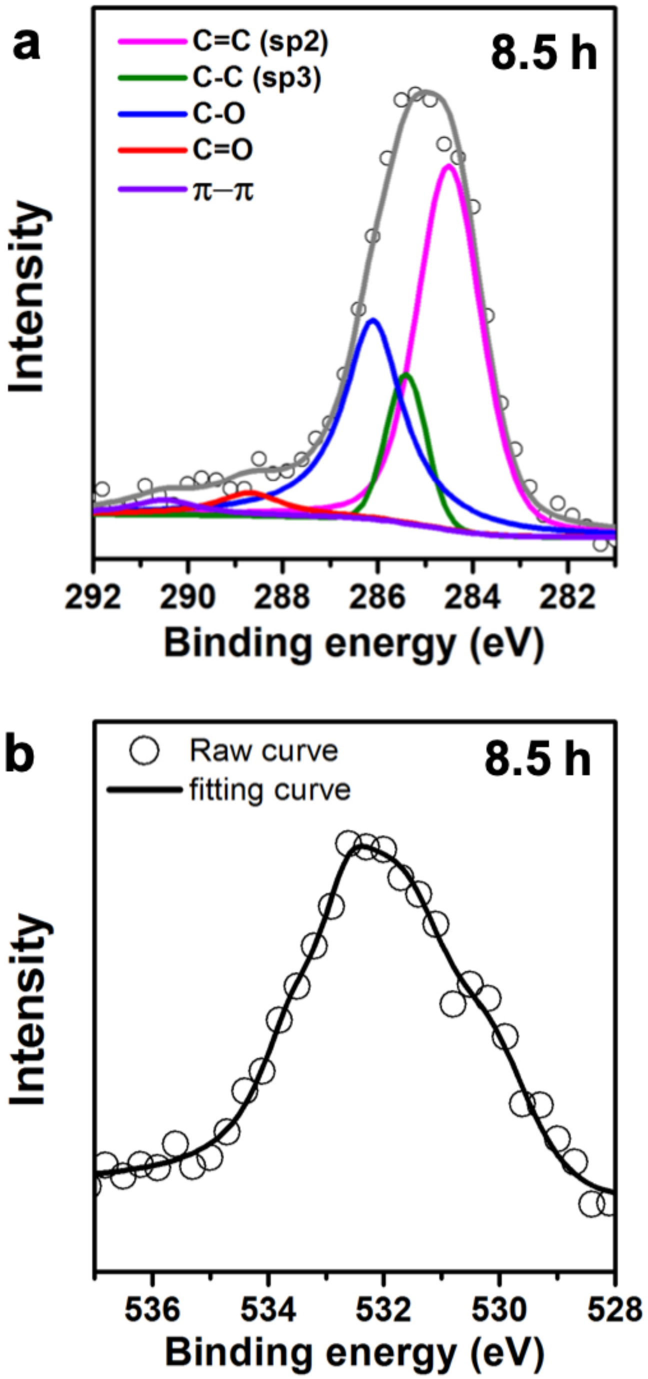

Supplementary Figure 11. **a** Deconvoluted carbon 1s spectra and **b** obtained oxygen 1s spectra of OCNTs reacted for 8.5 h.

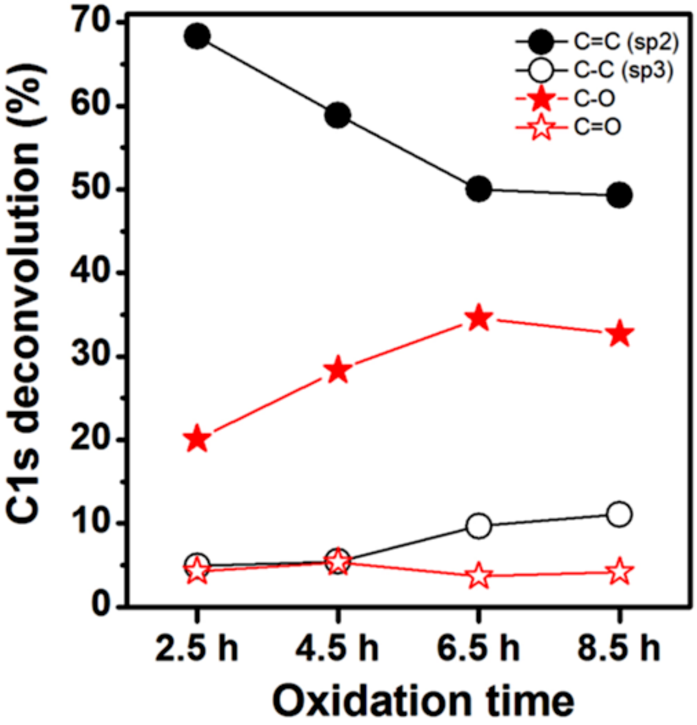

Supplementary Figure 12. Deconvoluted carbon 1s peak result of OCNTs reacted from 2.5 h to 8.5 h.

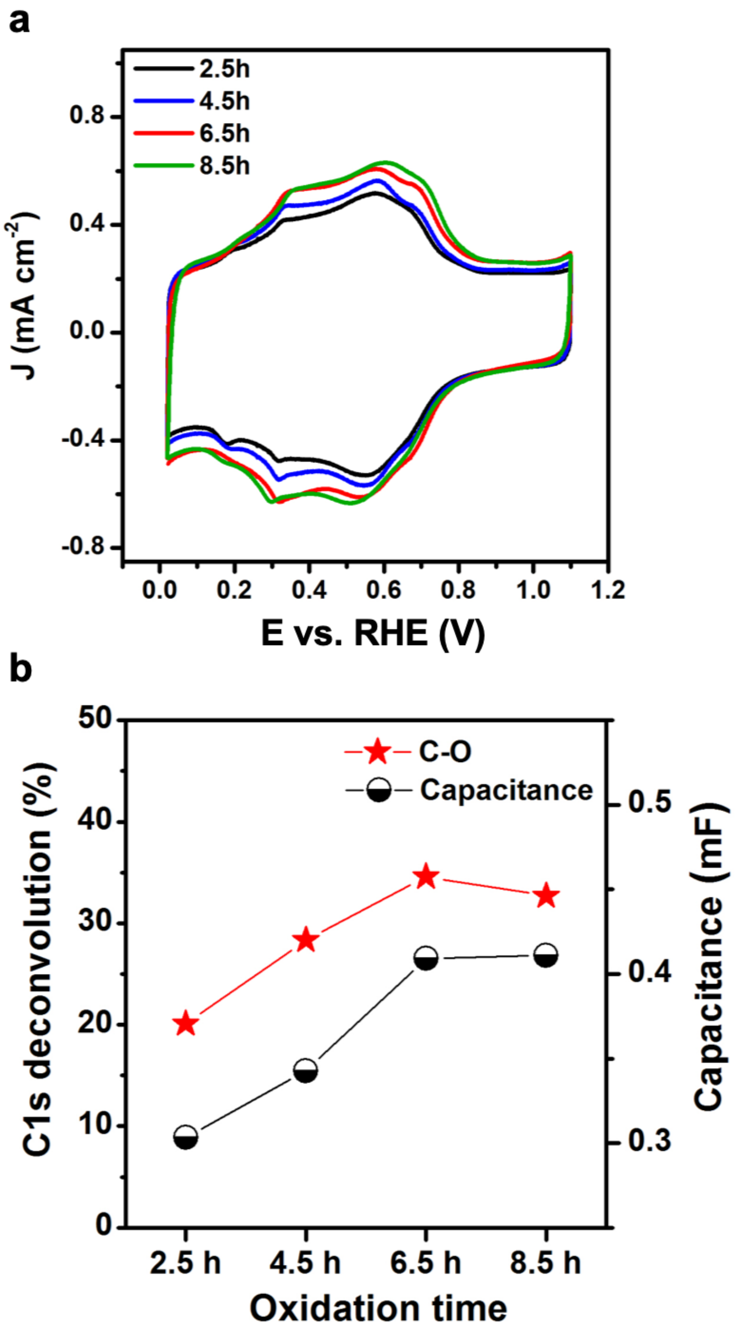

Supplementary Figure 13. **a** Cyclic voltammogram curves of OCNT samples in Ar-saturatred 0.1 M $HClO_4$ with a scan rate of 50 mV s^{-1}. **b** Calculated relative ratios of C-O groups and the capacitance of redox peaks from CV for different OCNT samples.

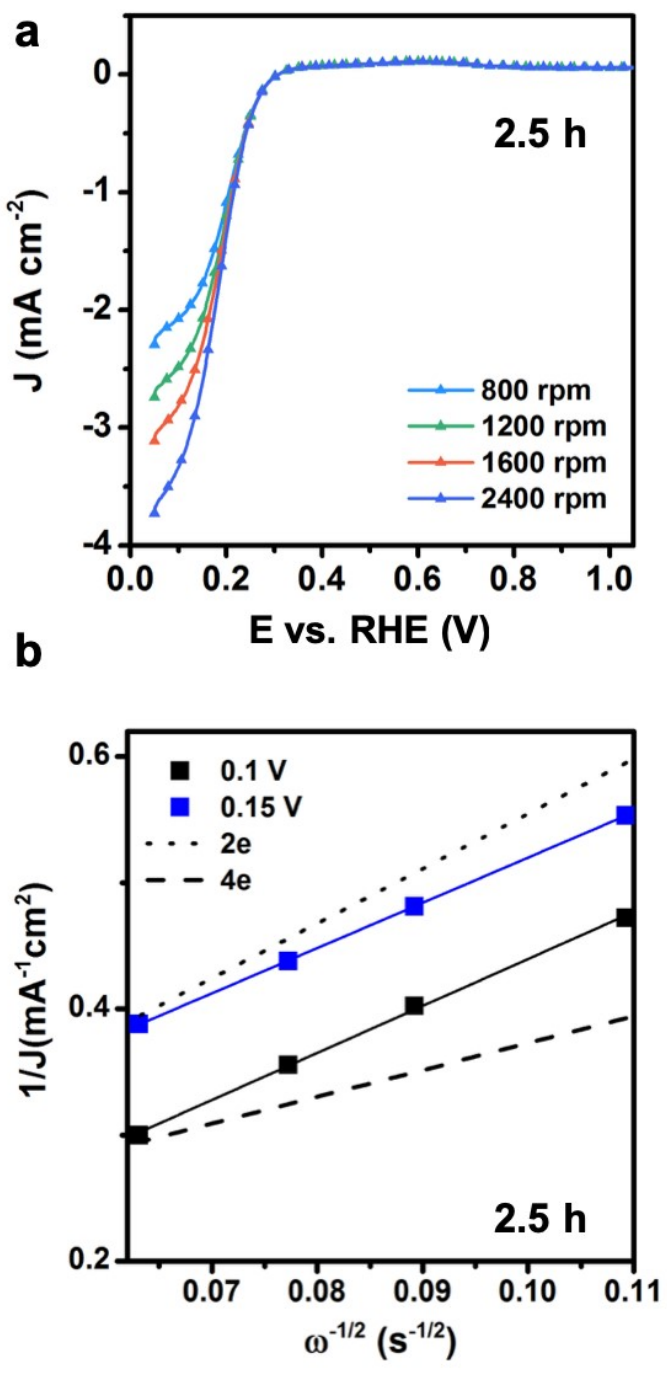

Supplementary Figure 14. **a** Polarization curves at various rotation rates in oxygen-saturated 0.1 M $HClO_4$ of and **b** Koutecky−Levich plot for the calculation of the transfer electron numbers of 2.5 h OCNT. The scan rate was 10 mV s^{-1} without IR correction. All experiments were performed at 25 ^{o}C. All the currents were normalized to the geometrical area of rotating disk electrode.

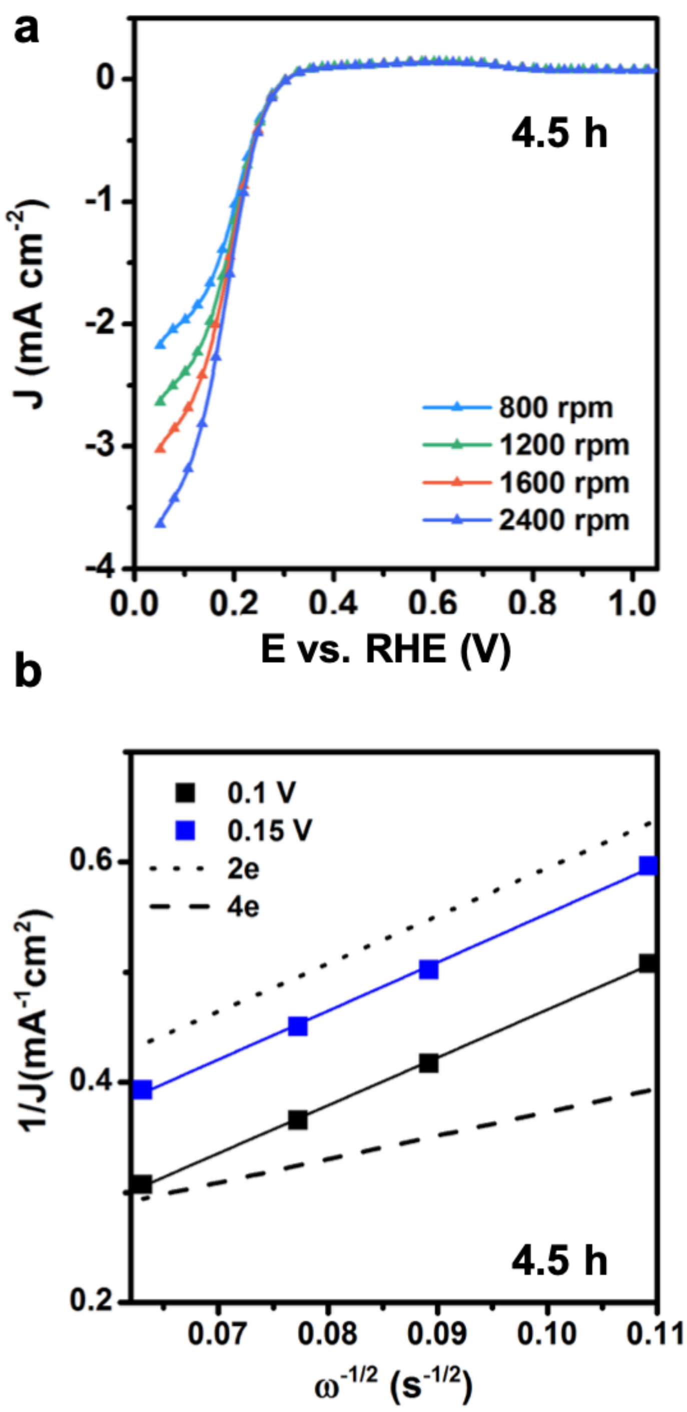

Supplementary Figure 15. **a** Polarization curves at various rotation rates in oxygen-saturated 0.1 M $HClO_4$ of and **b** Koutecky−Levich plot for the calculation of the transfer electron numbers of 4.5 h OCNT. The scan rate was 10 mV s^{-1} without IR correction. All experiments were performed at 25 ºC. All the currents were normalized to the geometrical area of rotating disk electrode.

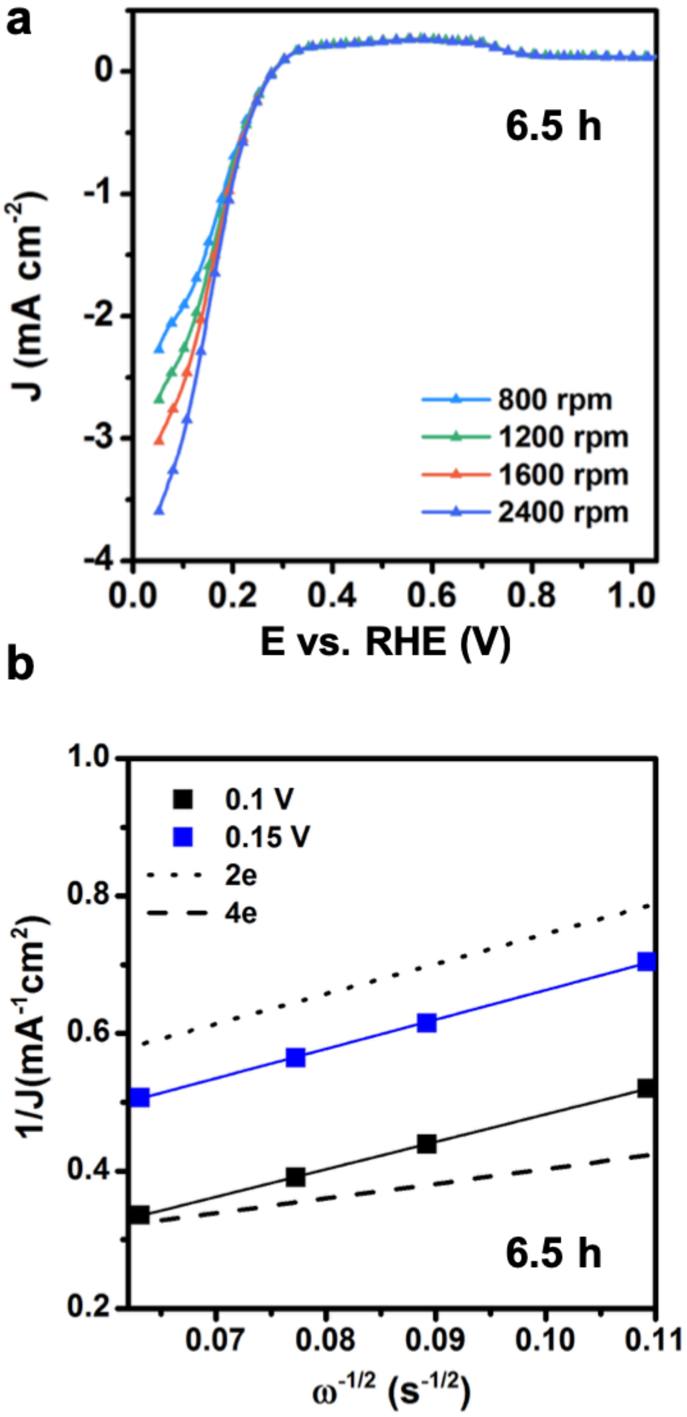

Supplementary Figure 16. **a** Polarization curves at various rotation rates in oxygen-saturated 0.1 M $HClO_4$ of and **b** Koutecky−Levich plot for the calculation of the transfer electron numbers of 6.5 h OCNT. The scan rate was 10 mV s^{-1} without IR correction. All experiments were performed at 25 ºC. All the currents were normalized to the geometrical area of rotating disk electrode.

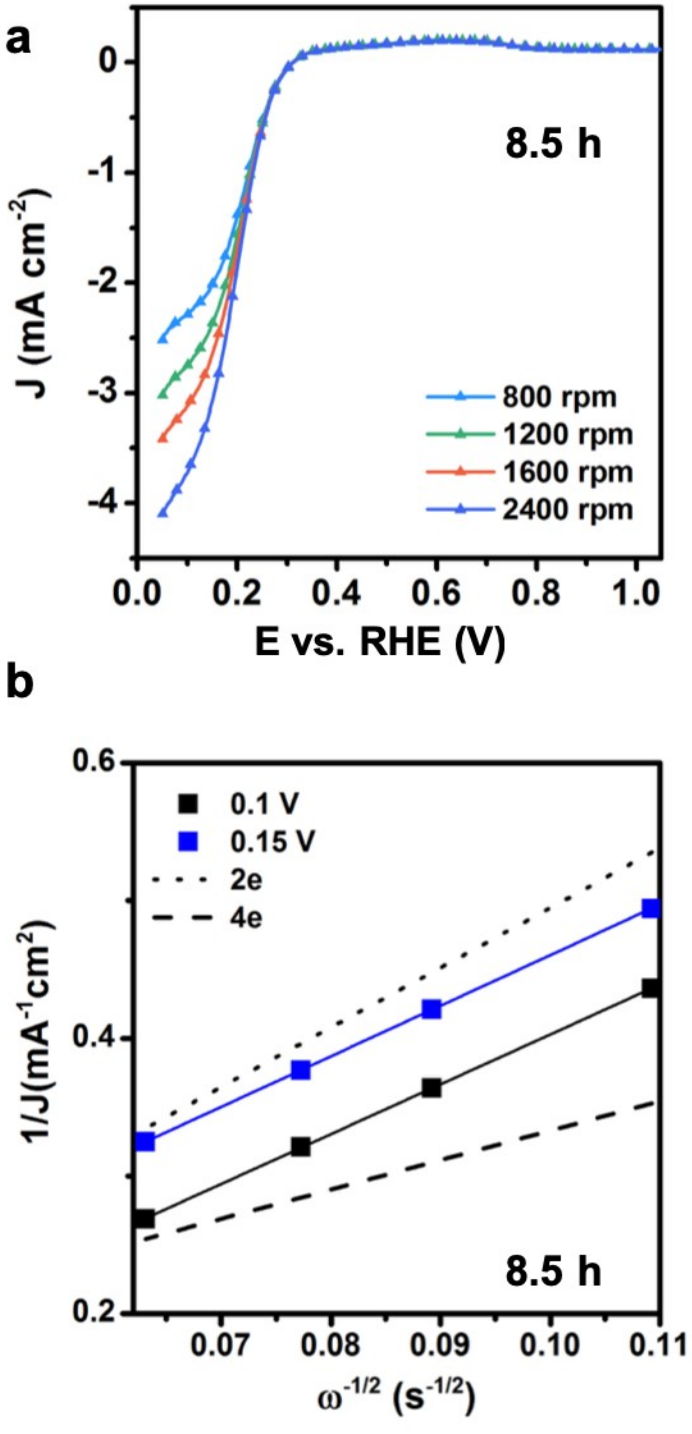

Supplementary Figure 17. **a** Polarization curves at various rotation rates in oxygen-saturated 0.1 M $HClO_4$ of and **b** Koutecky−Levich plot for the calculation of the transfer electron numbers of 8.5 h OCNT. The scan rate was 10 mV s^{-1} without IR correction. All experiments were performed at 25 °C. All the currents were normalized to the geometrical area of rotating disk electrode.

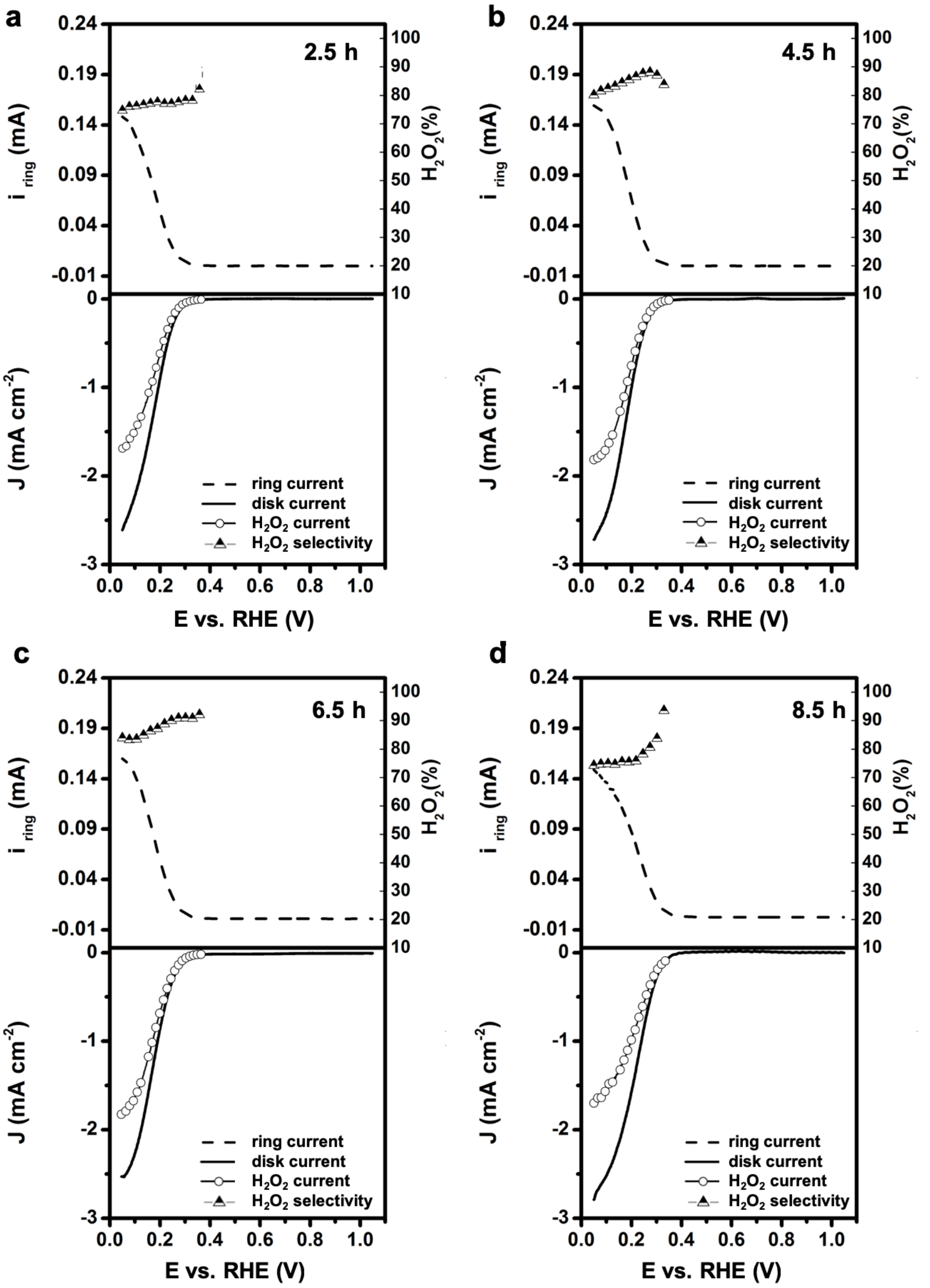

Supplementary Figure 18. RRDE voltammograms in O_2-saturated $HClO_4$ electrolyte with a scan rate of 10 mV s^{-1} at 1600 rpm (only the anodic cycle is shown). The disc current, ring current, hydrogen peroxide current calculated from the ring current and selectivity during the reaction of OCNT samples reacted from **a** 2.5 h, **b** 4.5 h, **c** 6.5 h, **d** 8.5 h.

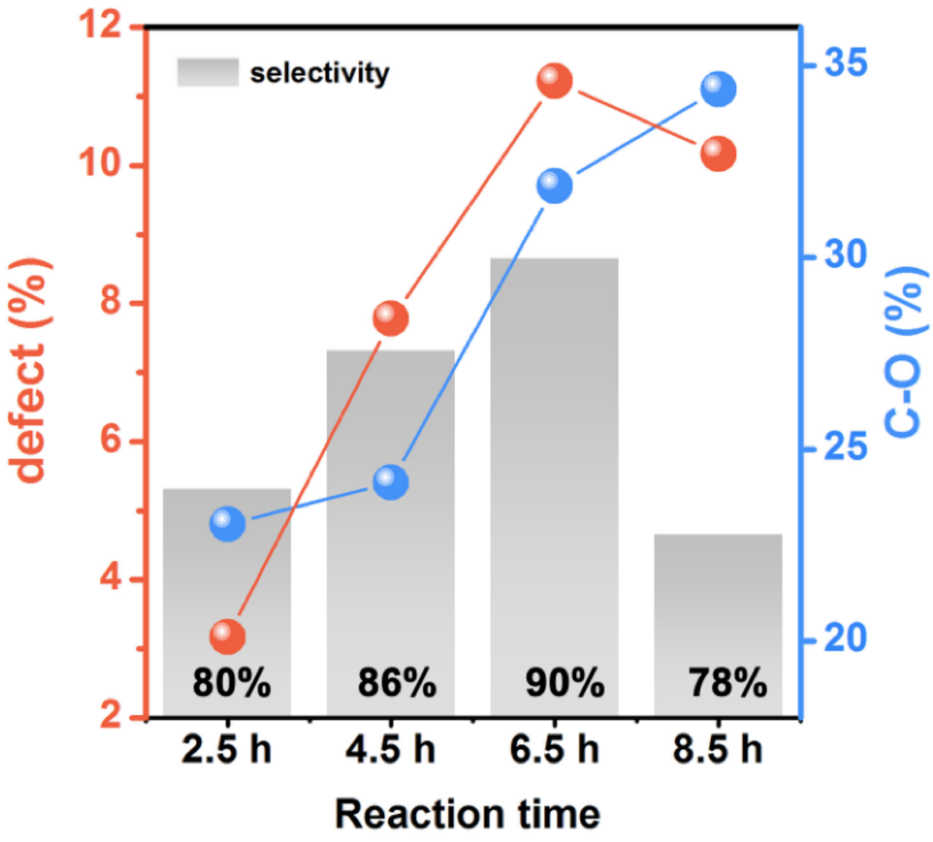

Supplementary Figure 19. Summary of the relationship between the ratios of defects, C-O groups in OCNT samples reacted from 2.5 h to 8.5 h and its selectivity toward $2e^-$ ORR.

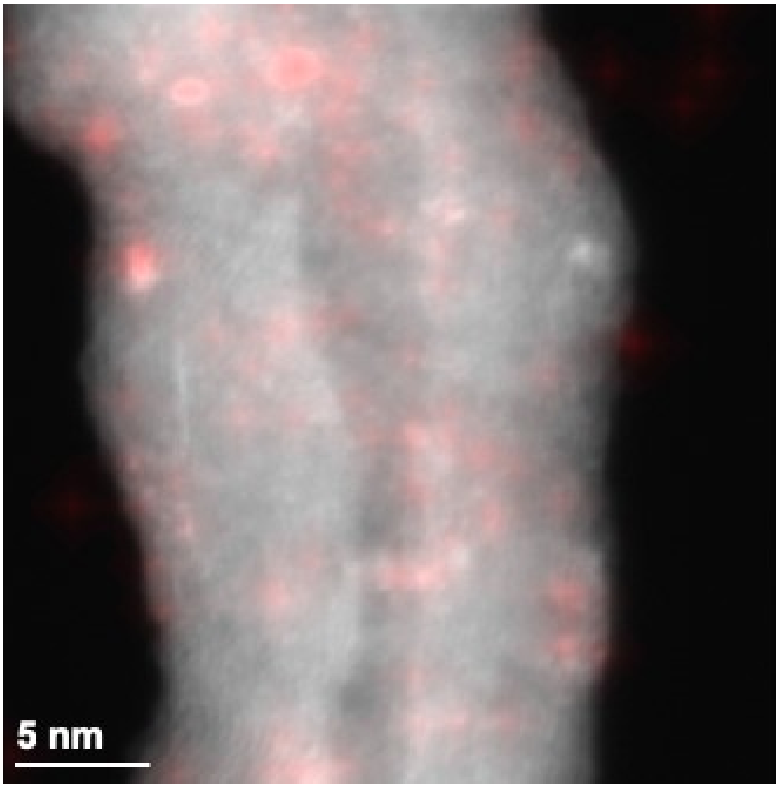

Supplementary Figure 20. Energy-dispersive X-ray spectroscopy (EDS) Pd element mapping of $Pd^{\delta+}$-OCNT.

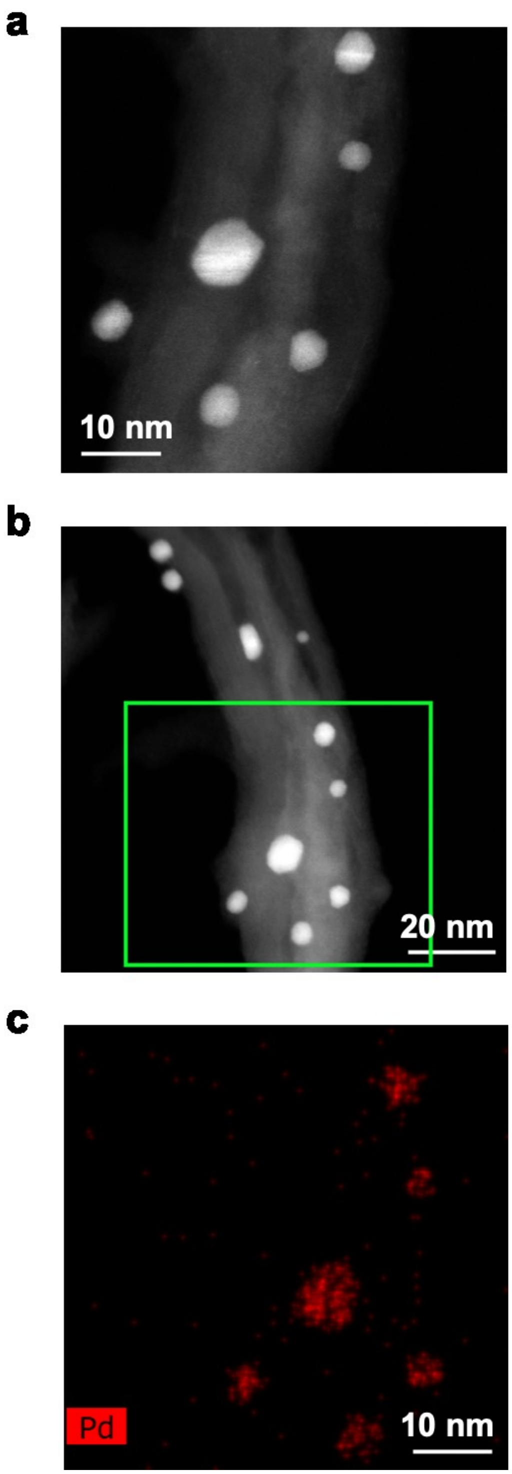

Supplementary Figure 21. **a** Annular dark-field (ADF)- scanning transmission electron of microscope (STEM) image of H-Pd-OCNT. **b** ADF-STEM image and **c** its corresponding energy-dispersive X-ray spectroscopy (EDS) Pd element mapping of H-Pd-OCNT.

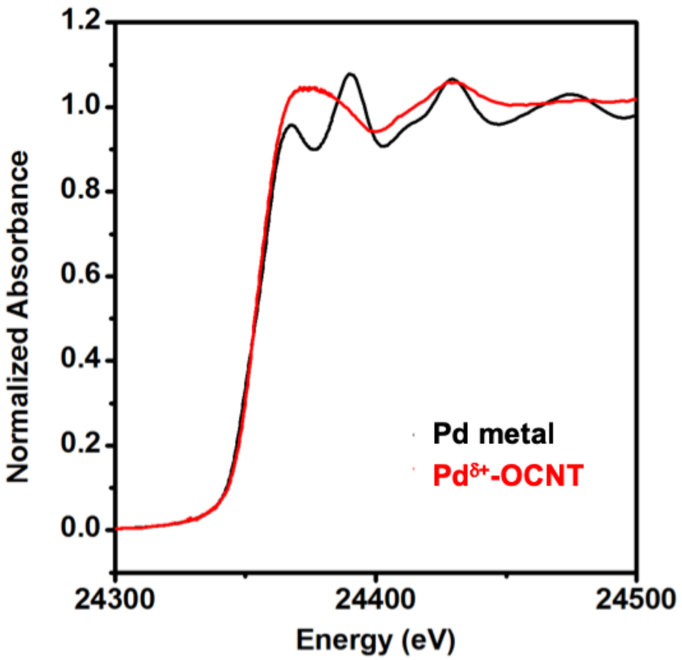

Supplementary Figure 22. X-ray absorption near-edge structure (XANES) analysis of $Pd^{\delta+}$-OCNT and Pd metal.

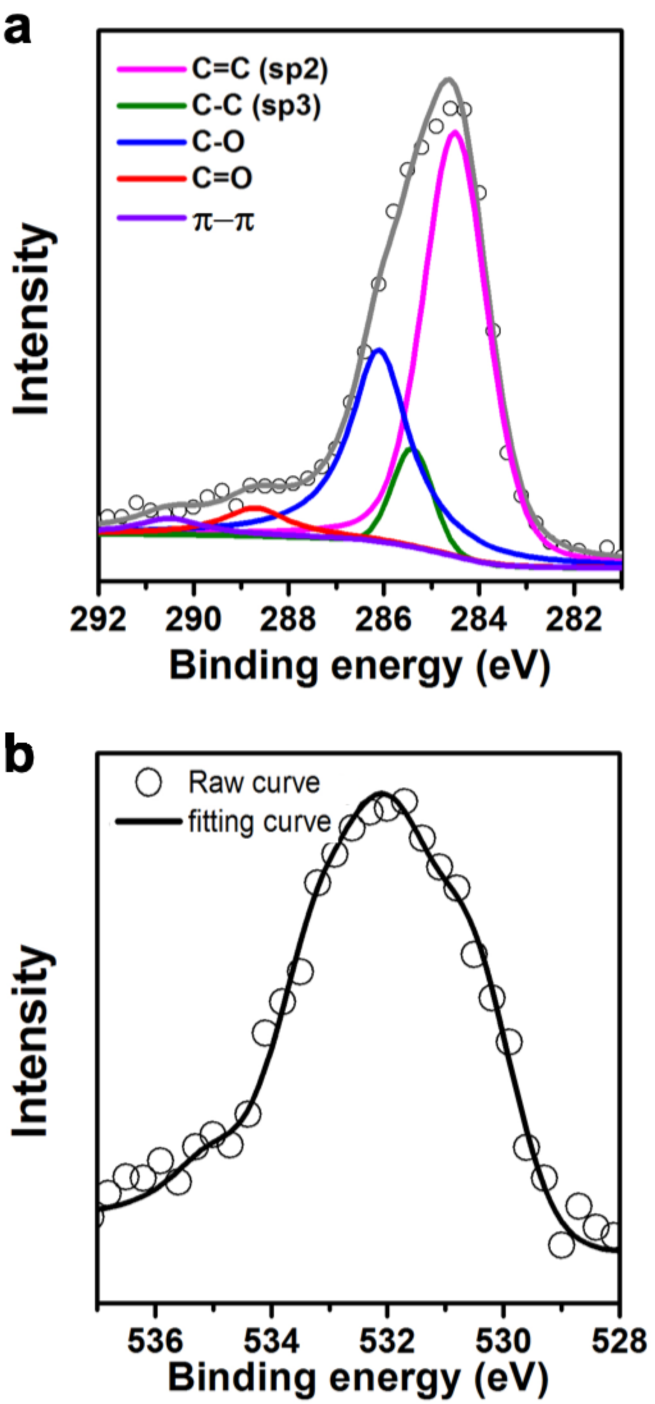

Supplementary Figure 23. **a** Deconvoluted carbon 1s and **b** oxygen 1s spectra of $Pd^{\delta+}$-OCNT.

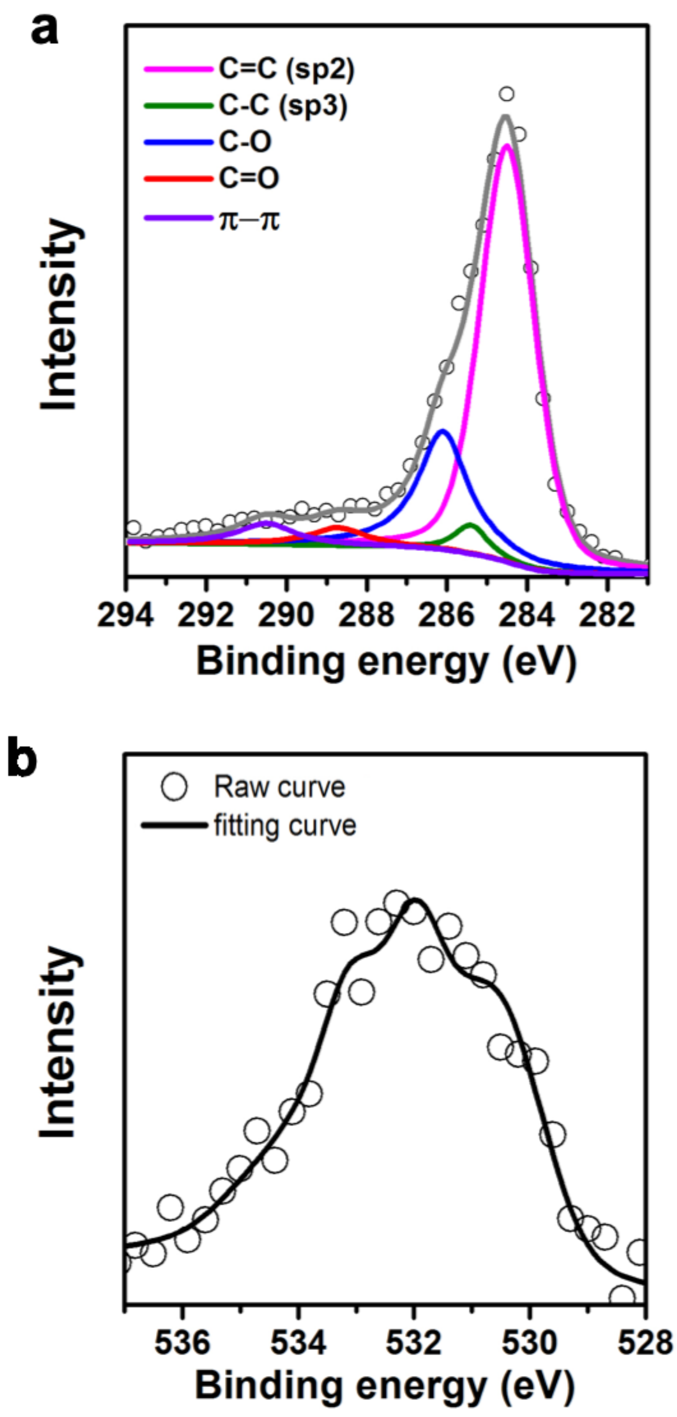

Supplementary Figure 24. **a** Deconvoluted carbon 1s and **b** oxygen 1s spectra of H-Pd-OCNT.

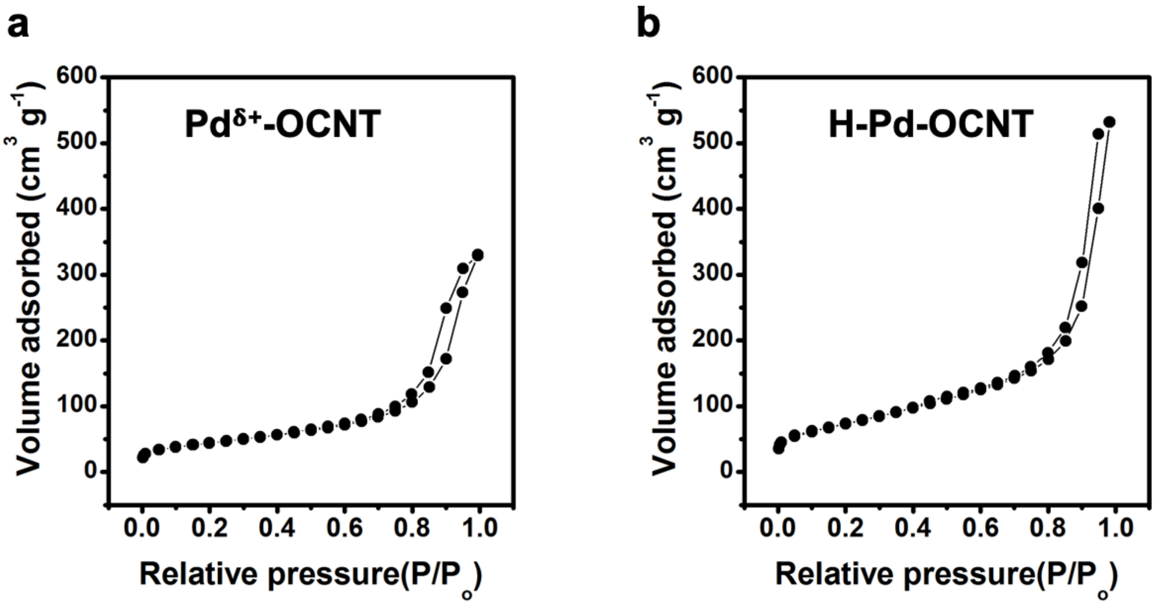

Supplementary Figure 25. The N_2 adsorption-desorption isotherms of **a** $Pd^{\delta+}$-OCNT and **b** H-Pd-OCNT.

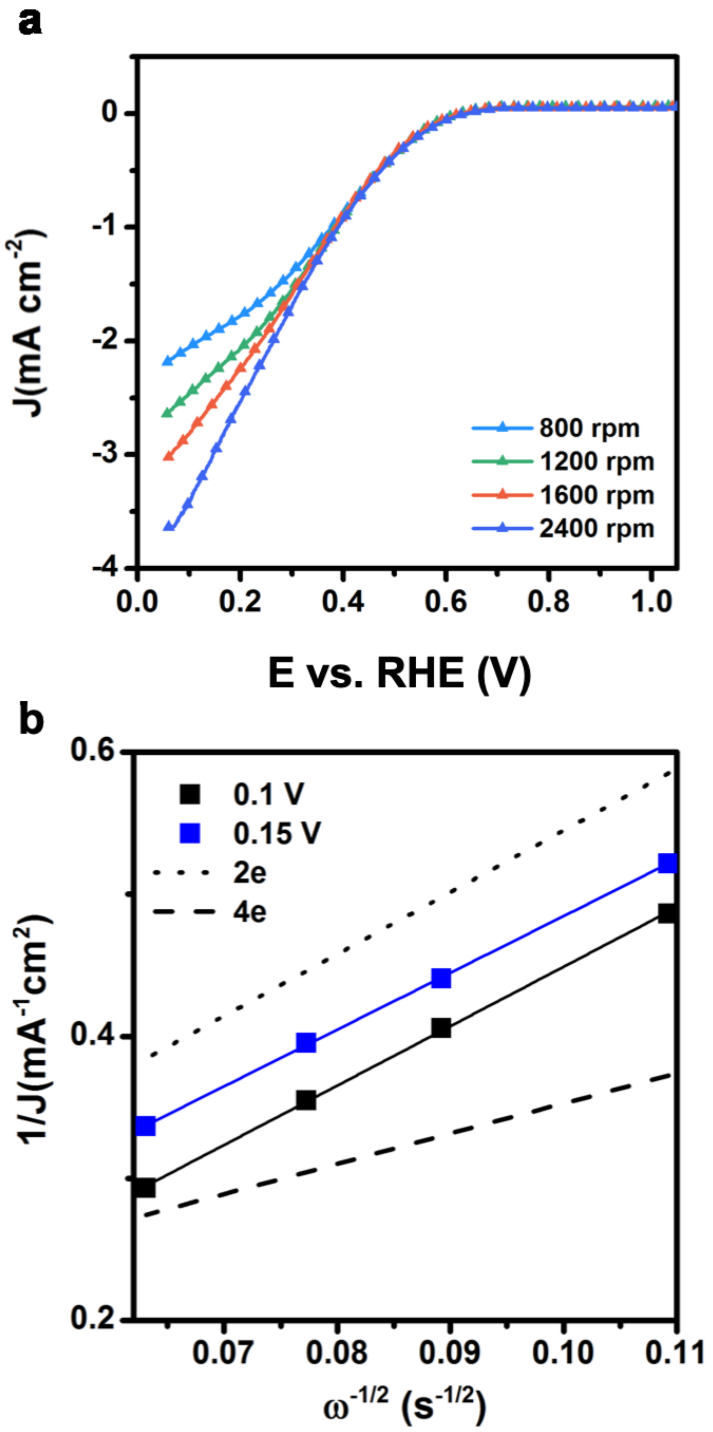

Supplementary Figure 26. **a** Polarization curves at various rotation rates in oxygen-saturated 0.1 M $HClO_4$ of and **b** Koutecky−Levich plot for the calculation of the transfer electron numbers of $Pd^{\delta+}$-OCNT. The scan rate was 10 mV s^{-1} without IR correction. All experiments were performed at 25 °C. All the currents were normalized to the geometrical area of rotating disk electrode.

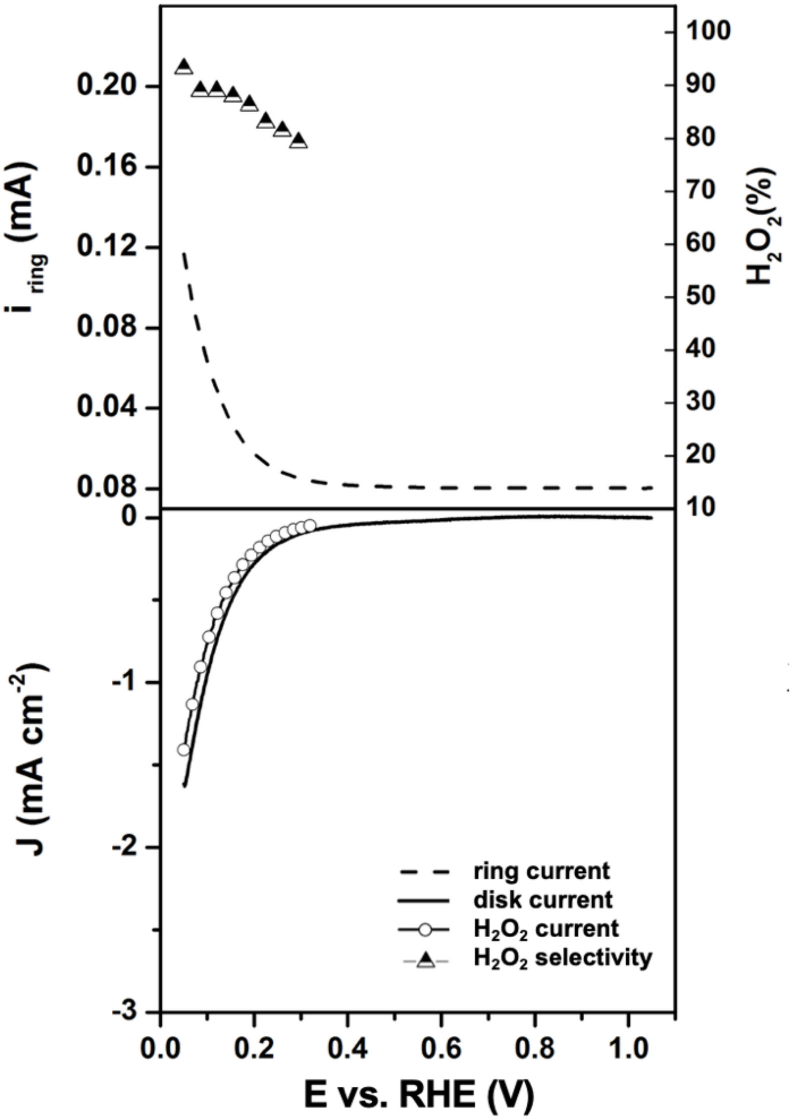

Supplementary Figure 27. RRDE voltammograms in O_2-saturated $HClO_4$ electrolyte with a scan rate of 10 mV s^{-1} at 1600 rpm (only the anodic cycle is shown). The disc current, ring current, hydrogen peroxide current calculated from the ring current and selectivity during the reaction of H-OCNT sample.

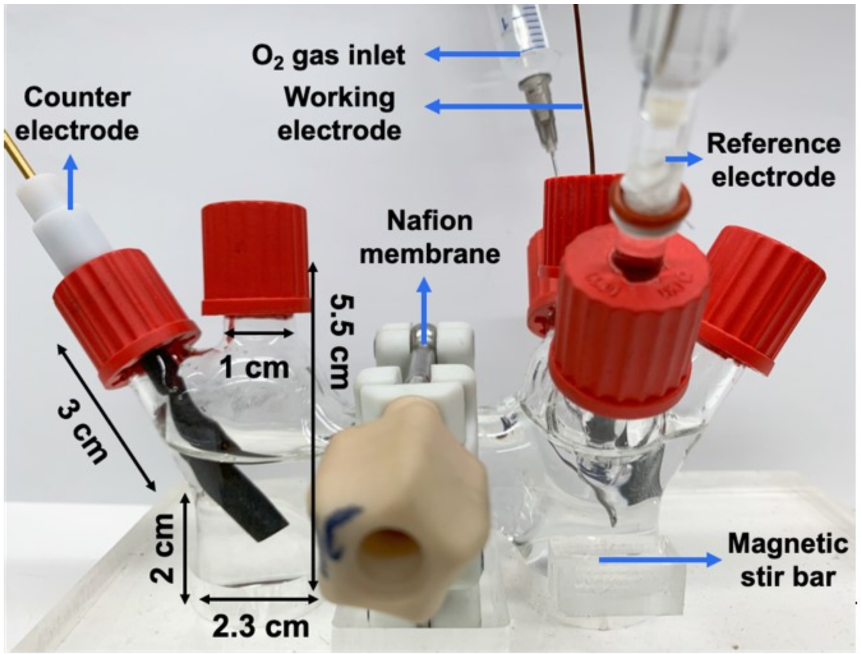

Supplementary Figure 28. The digital photo image of the H-cell used for the H_2O_2 yield test. The electrolyte volume in the working electrode part was 17 ml and the total volume of this part was 30 ml.

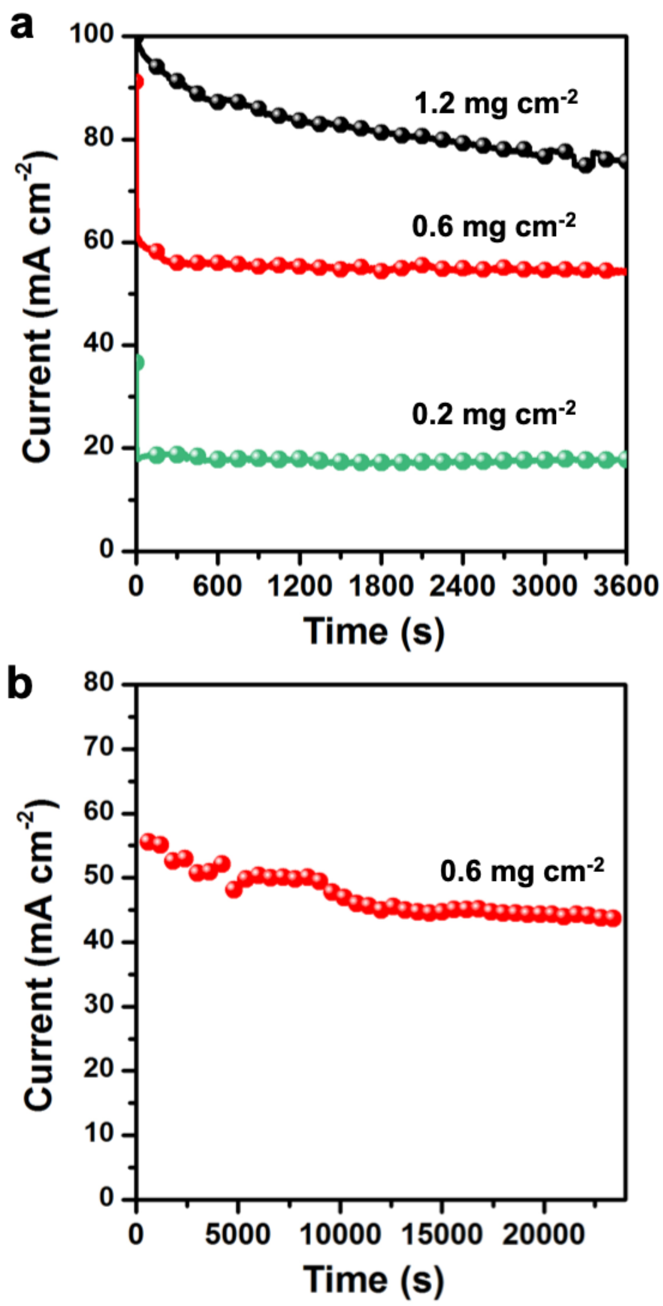

Supplementary Figure 29. Stability test in H-cell. **a** Current densities obtained from H-cell test by adjusting the mass loadings of electrocatalyst at 0.1 V. **b** H-cell stability test with a electrocatalyst mass loading of 0.6 mg cm^{-2} at 0.1 V.

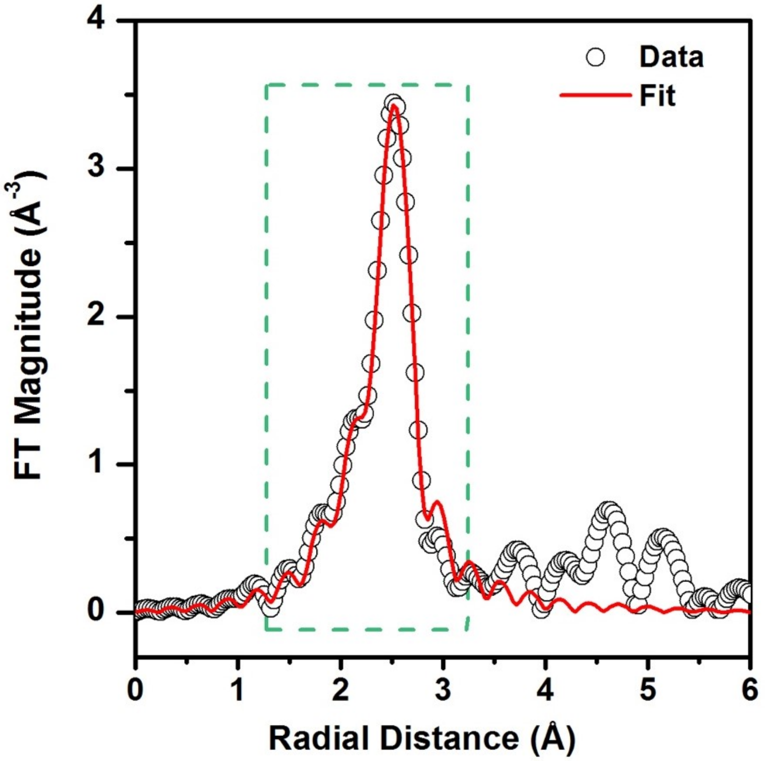

Supplementary Figure 30. FT-transformed EXAFS analysis of Pd K-edge data for Pd foil.

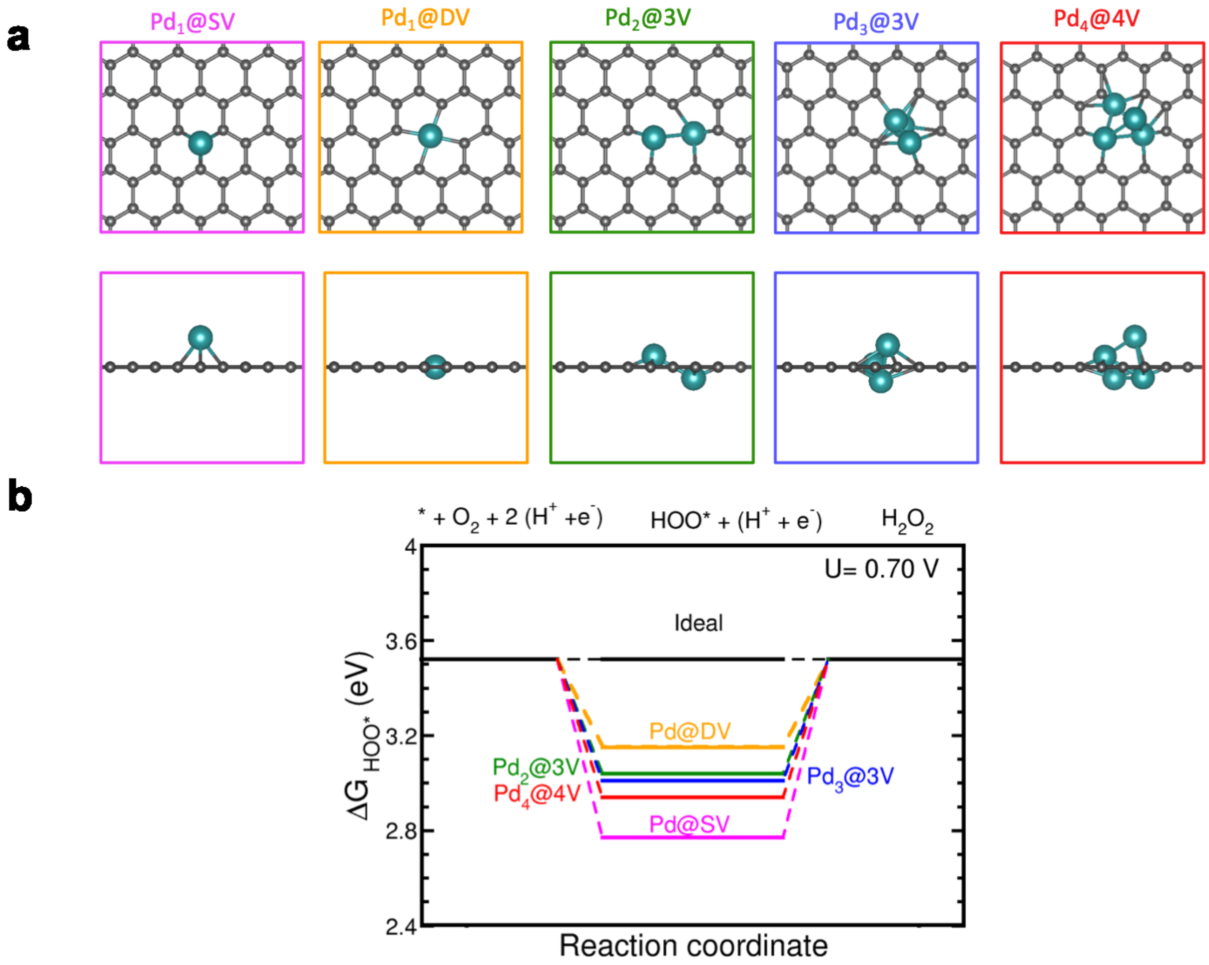

Supplementary Figure 31. **a** Optimized DFT model structures for Pd_n clusters embedded in graphene defects. Color code; C: gray, Cyan: Pd. **b** Free energy diagram for 2e-ORR over different model structures presented in **a** at standard redox potential (0.70 V).

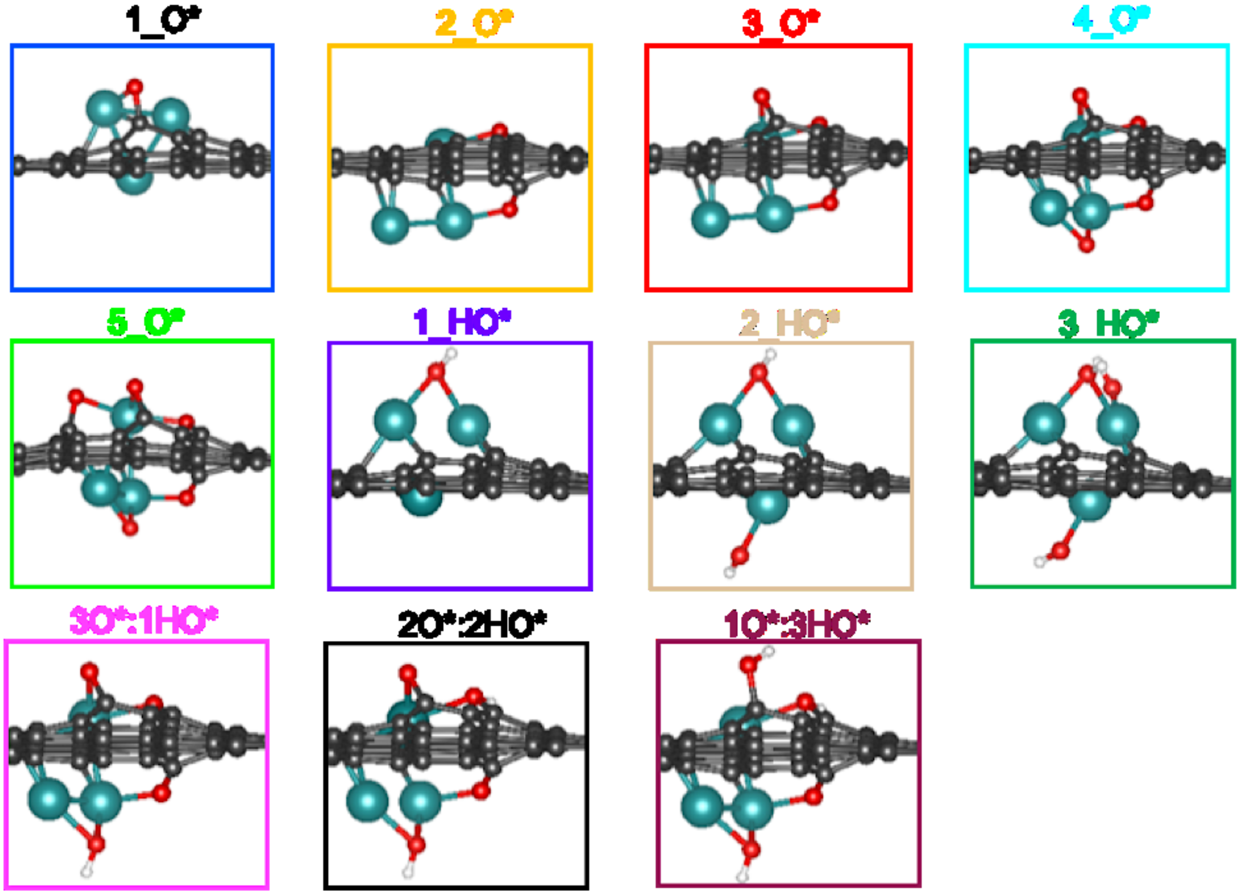

Supplementary Figure 32. Side views of the most stable coverages examined for different oxygenated species on Pd_3@3V. The line colors match the lines in the Pourbiax diagram presented in Figure 5a of the main manuscript. Color code; C: gray, O: red, Cyan: Pd.

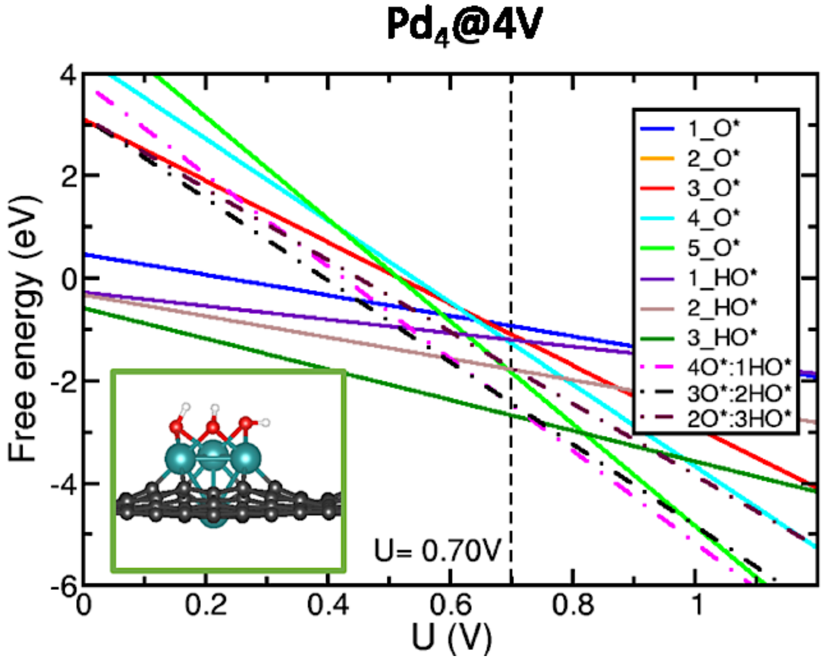

Supplementary Figure 33. Pourbiax diagram for investigating the steady state coverage of the oxygenated species on the Pd_4@4V. The inset shows the side views of the most stable coverage.

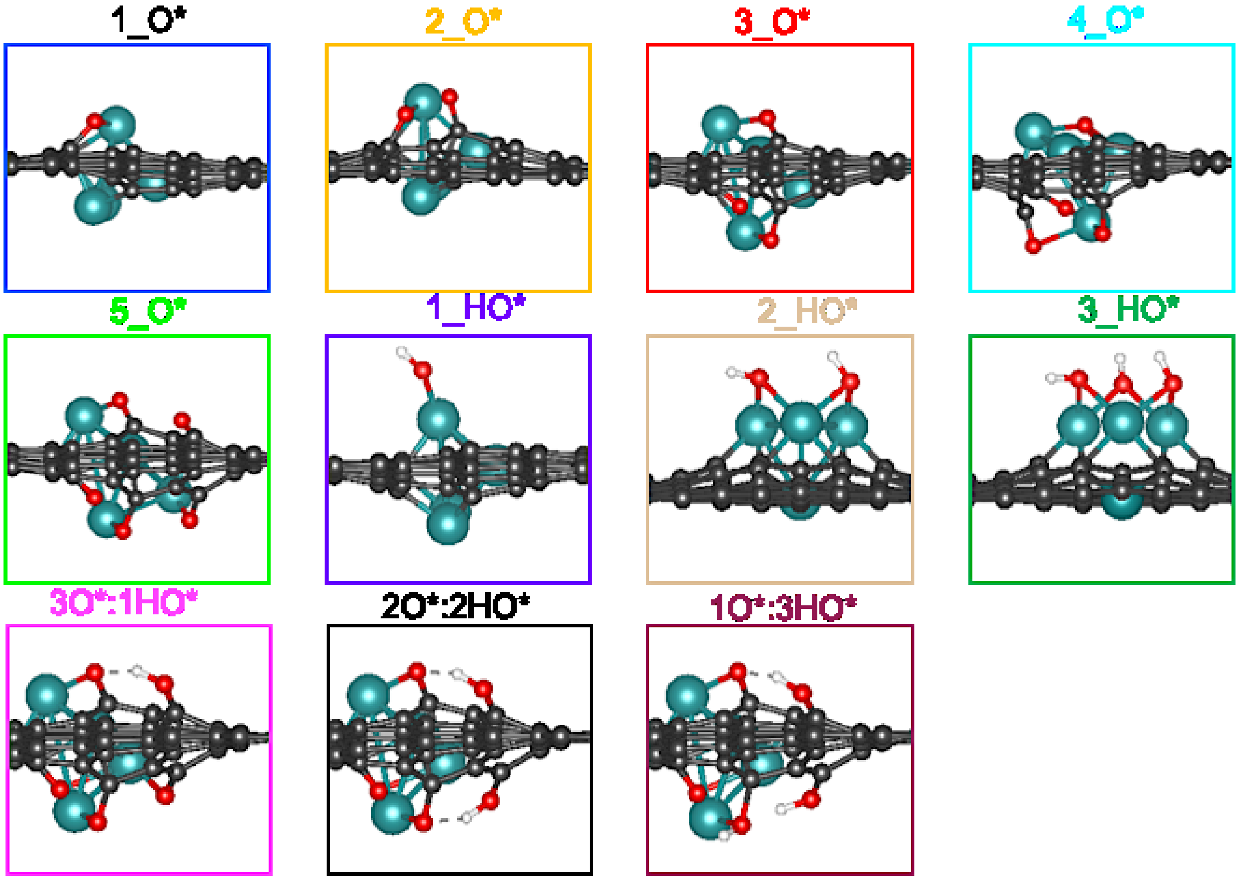

Supplementary Figure 34. Side views of the most stable coverages examined for different oxygenated species on Pd_4@4V. The line colors match the lines in the Pourbiax diagram presented in Supplementary Figure 33.

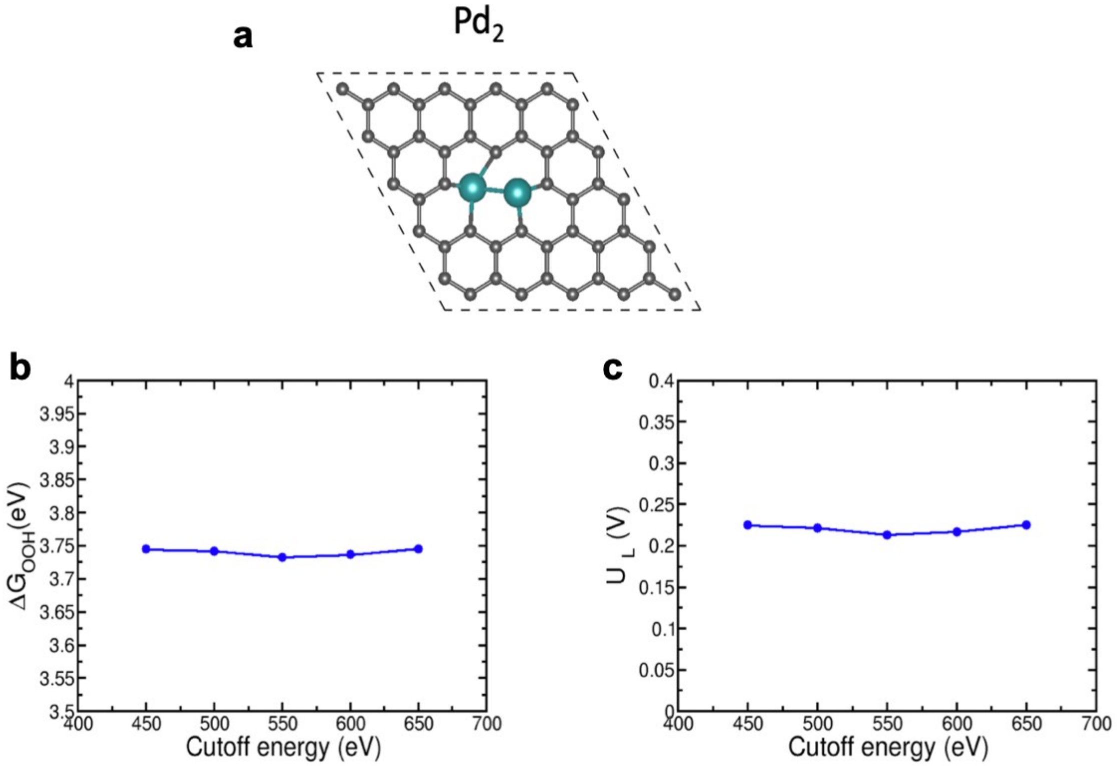

Supplementary Figure 35. **a** Top figure shows the Pd_2 cluster embedded at graphene vacancy in a 5×5 unit cell. **b** and **c** are the cutoff energy convergency plots for the calculated adsorption energy of OOH* (our activity descriptor) and calculated limiting potential, respectively.

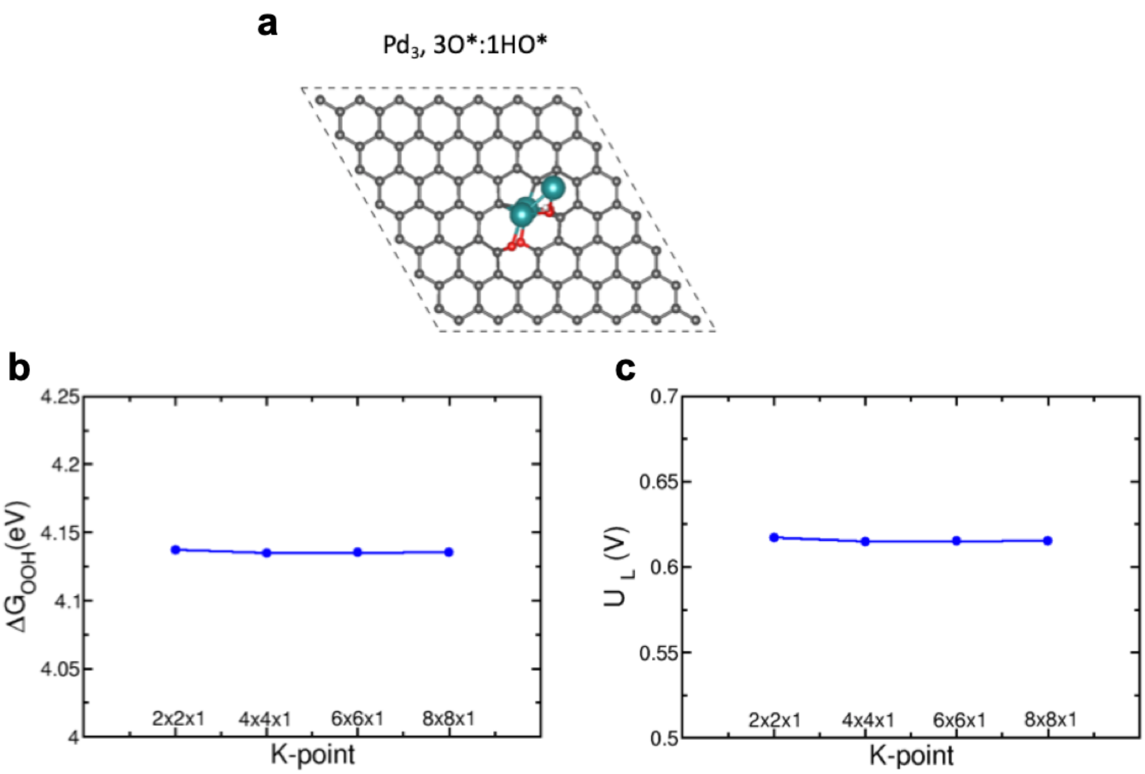

Supplementary Figure 36. **a** Top figure shows the optimized structure with the most stable coverage for Pd_3 cluster (3O*:1HO*) modeled in a 7×7 unit cell. **b** and **c** are the calculated adsorption energy of OOH* and calculated limiting potential for different K-point sampling, respectively.

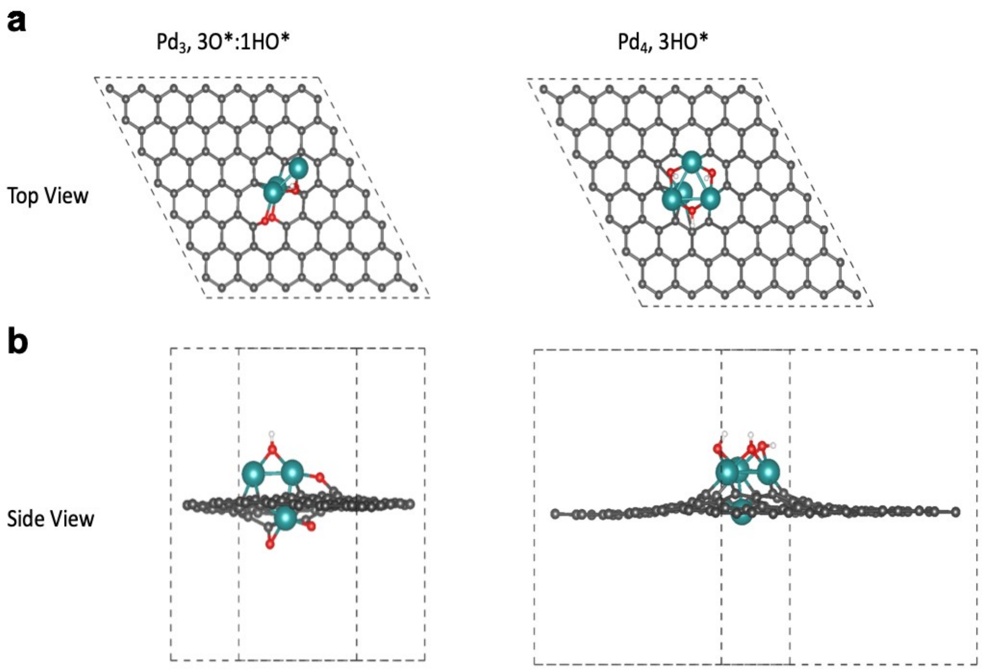

Supplementary Figure 37. **a** Top and **b** side views of the optimized structures for Pd_3 and Pd_4 clusters which were done in a 7x7 unite cell to avoid the interaction between periodic images. The side views show the angle we chose for the Pourbiax diagrams in the main manuscript and Supplementary Figure 33.

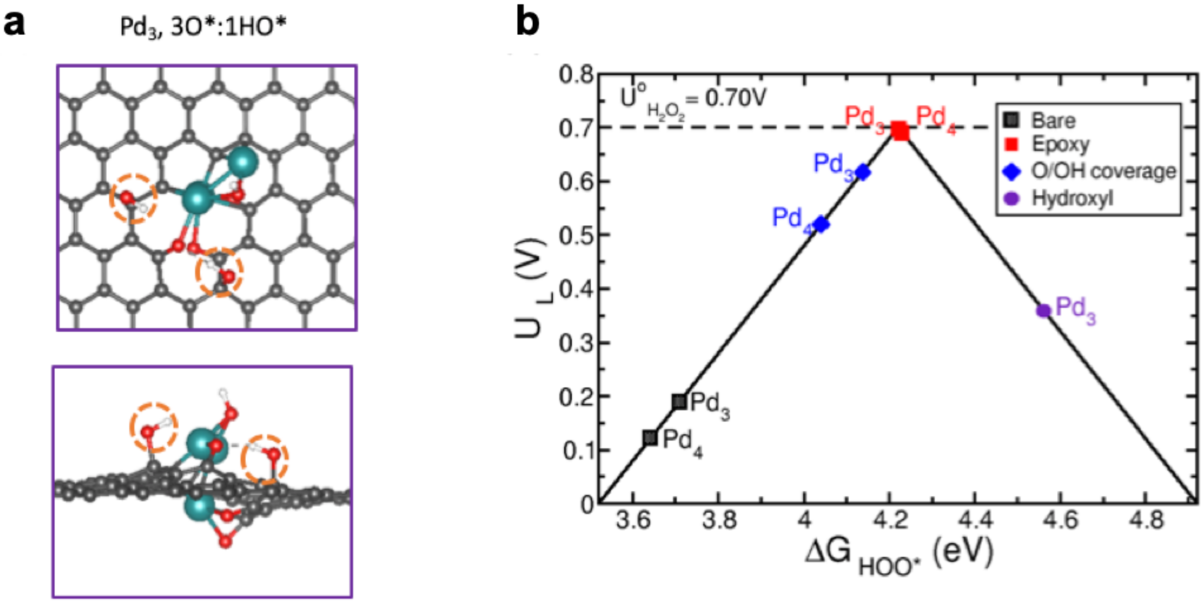

Supplementary Figure 38. **a** Optimized DFT model structures with nearby hydroxyl functional groups (highlighted by orange dashed circles) on the most stable O*/HO* covered Pd_3 clusters. **b** Activity volcano plot, including the hydroxyl functional group for the example of Pd_3 cluster.

Supplementary Tables

Supplementary Table 1. EXAFS fitting results.

Parameter	Entry		
	Pd Metal	H-Pd-OCNT	$Pd^{\delta+}$-OCNT
Independent Points	12.9267578	8.7089844	13.296875
Number of Variables	4	4	7
Reduced Chi-square	2185.0284425	523.9641474	126.9657377
R-factor	0.0042065	0.0138021	0.0270848
k-range ($Å^{-1}$)	3.000 – 13.931	2.488 – 11.601	2.015 – 11.296
R-range (Å)	1.3 – 3.2	1.761 – 3.3	1.1 – 3.4
Number of Data Set	1	1	1
Structure Model 1			
Chemical Fomula	Pd		
Space Group	Fm-3m		
Lattice Constant	3.900 Å		
Structure Model 2			
Chemical Fomula	PdO		
Space Group	$P4_2/mmc$		
Lattice Constant	a=b=3.096 Å, c=5.442 Å		

Supplementary Table 2. EXAFS fitting parameter of Pd metal.

Path	Coordination Number [a]	E_0 (eV)	R (Å)	σ^2 ($Å^2$)	Remarks
Pd-Pd	12	3.4(3)	2.733(6)	0.0055(2)	Pd

[a] Amplitude reduction factor was attained from this fitting. All the fitting was done in R-space

Supplementary Table 3. EXAFS fitting parameter of H-Pd-OCNT.

Path	Coordination Number [a]	E_0 (eV)	R (Å)	σ^2 (Å^2)	Remarks
Pd-Pd	7.3(9)	2.4(8)	2.733(6)	0.0058(9)	Pd

[a] Amplitude reduction factor was attained from the reference Pd metal foil. All the fitting was done in R-space

Supplementary Table 4. EXAFS fitting parameter of $Pd^{\delta+}$-OCNT.

Path	Coordination Number [a]	E_0 (eV)	R (Å)	σ^2 (Å^2)	Remarks
Pd-Pd	2.5(6)	0.29 [b]	2.743(6)	0.004(2)	Pd
Pd-O	2.7(4)	10(1)	2.11(2)	0.008(4)	PdO

[a] Amplitude reduction factor was attained from the reference Pd metal foil. All the fitting was done in R-space
[b] Fixed during the fitting.

Supplementary Table 5 Summary of the specific surface area and average pore diameters of $Pd^{\delta+}$-OCNT and H-Pd-OCNT.

Materials	Specific surface area (m^2 g^{-1})	Average pore diameter (nm)
$Pd^{\delta+}$-OCNT	163.4	12.5
H-Pd-OCNT	265.1	12.6

Supplementary Table 6 Summary of reported noble-metal based elecrtocatalysts for 2e ORR in the acid electrolyte.

Materials	selectivity	Onset potential	Mass activity ($A\ g^{-1}$) 0.55 V	Mass activity* ($A\ mg^{-1}$) 0.45 V	Literature
$Pd^{\delta+}$-OCNT	95%	0.70 V	597	1.946	This work
Pt/TiN	65%	~0.70 V	-	0.87	[2]
Pd_2Hg_5/C	~95%	~0.70 V	530	1.366	[3]
$PtHg_4/C$	~95%	~0.60 V	167	-	[4]
AuPd/C	80%	~0.70 V	-	-	[5]
Carbon-coated Pt nanoparticles	41%	~0.70 V	-	-	[6]

Supplementary Note 1. The effect of electrocatalyst mass loadings

We performed additional H-cell experiments with increased electrocatalyst mass loadings. Stable current densities of 19 and 55 mA cm^{-2} could be obtained when the electrocatalyst mass loadings were increased to 0.2 and 0.6 mg cm^{-2}, respectively. When the mass loading increased to 1.2 mg cm^{-2}, the thick catalyst layer (catalysts were deposited on a relatively small area of ~0.49 cm^{-2} due to the size limit of the chamber) cracked more easily and the catalysts tended to feel off the electrode during the test due to the large O_2 flux, resulting in current density decay from 100 to 78 mA cm^{-2} during the 1 h operation.

Supplementary Note 2. The effect of other oxygen functional groups

We have explored the effect of a range of different oxygen functional groups including hydroxyl, carbonyl and etheric groups. Among all these functional groups, we found epoxy groups to have the most meaningful impact on the ΔG_{HOO*} which well aligned with the experimental results. Supplementary Figure 38 displays the activity volcano including an example of the effect of hydroxyl group on the adsorption energy of HOO* and calculated limiting potential for Pd_3 covered with 3O*:1HO*. The results show that similar to epoxy group, including the hydroxyl functional group weakens the adsorption energy of HOO*. This in turn results in increasing the selectivity toward H_2O_2, [1]however, the calculated limiting potential is not significantly high.

Supplementary References

1 Chen, S. *et al.* Defective carbon-based materials for the electrochemical synthesis of hydrogen peroxide. *ACS Sustain. Chem. Eng.* **6**, 311-317 (2018).

2 Yang, S., Kim, J., Tak, Y. J., Soon, A. & Lee, H. Single - atom catalyst of platinum supported on titanium nitride for selective electrochemical reactions. *Angew. Chem. Int. Ed.***55**, 2058-2062 (2016).

3 Verdaguer-Casadevall, A. *et al.* Trends in the electrochemical synthesis of H_2O_2: enhancing activity and selectivity by electrocatalytic site engineering. *Nano Lett.* **14**, 1603-1608 (2014).

4 Siahrostami, S. *et al.* Enabling direct H_2O_2 production through rational electrocatalyst design. *Nat. Mater.* **12**, 1137-1142 (2013).

5 Pizzutilo, E. *et al.* Electrocatalytic synthesis of hydrogen peroxide on Au-Pd nanoparticles: From fundamentals to continuous production. *Chem. Phys. Lett.* **683**, 436-442 (2017).

6 Choi, C. H. *et al.* Hydrogen peroxide synthesis via enhanced two-electron oxygen reduction pathway on carbon-coated Pt surface. *J. Phys. Chem. C* **118**, 30063-30070 (2014).
